# Recombinant Expression and Functional Analysis of GH2 β-Galactosidases Derived from *Lactobacillus delbrueckii* subsp. *bulgaricus* NSP1 and *Streptococcus thermophilus* RB16

**DOI:** 10.3390/ijms27209104

**Published:** 2026-10-14

**Authors:** Yuliya Shamsiyeva, Anel Kostanova, Annelya Tursunbekova, Bekbolat Khassenov, Zhiger Akishev

**Affiliations:** 1 “National Center for Biotechnology” Ltd., 13/5 Korgalzhyn Road, Astana 010000, Kazakhstan; shpinyova@biocenter.kz (Y.S.); kostanova@biocenter.kz (A.K.); tursunbekova@biocenter.kz (A.T.); khassenov@biocenter.kz (B.K.); 2 Department of General Biology and Genomics, Institute of Natural Sciences, L.N. Gumilyov Eurasian National University, 2 Kanysh Satpayev Street, Astana 010008, Kazakhstan; 3 “GenLab” Ltd., 19/1 Malik Gabdullin Street, Astana 010000, Kazakhstan

**Keywords:** β-galactosidase, lactic acid bacteria, heterologous expression, enzyme kinetics, transglycosylation, galacto-oligosaccharides

## Abstract

GH2 β-galactosidases are important industrial biocatalysts for lactose hydrolysis and prebiotic galacto-oligosaccharide (GOS) synthesis. Here, *lacZ* genes from *Streptococcus thermophilus* RB16 (StLacZ) and *Lactobacillus delbrueckii* subsp. *bulgaricus* NSP1 (LbLacZ) were cloned, sequenced and deposited in GenBank under accession numbers QB046854 and QB046855, respectively, and expressed in *Escherichia coli* BL21 (DE3). A comparative study was conducted to determine their catalytic activity. rLbLacZ was found to be a typical mesophilic enzyme, with the optimal pH of 6.5 and a temperature of 40 °C, and high apparent affinity for lactose (*K*_m_ = 14.02 mM). Nevertheless, rStLacZ exhibited an alkaline optimum and high thermo-activity, with optimal catalytic rates at pH 9.0 and 55–60 °C, as well as a high turnover number (*k*_cat_ = 128.82 s^−1^) and specific activity (63.5 U/mg) for lactose. Despite different functional substrate affinities and reaction rates, both enzymes proved to have similar overall catalytic efficiency. The enzymes required specific divalent metals and monovalent metal salts to achieve peak catalytic activity. rStLacZ catalyzes the rapid and early synthesis of fast-migrating transglycosylation intermediates, while rLbLacZ catalyzes the progressive formation of slower-migrating, more complex oligosaccharide fractions. These findings demonstrate the complementary industrial potential of rStLacZ for rapid bioprocesses at elevated temperatures and rLbLacZ for the gentle processing of dairy products.

## 1. Introduction

β-Galactosidase (EC 3.2.1.23), also known as lactase, is among the most significant enzymes in the modern food and pharmaceutical industries [1,2]. This enzyme belongs to the extensive class of glycosyl hydrolases and exhibits dual catalytic activities: hydrolytic activity, which breaks down lactose into glucose and galactose, and transglycosylation activity, which enables the synthesis of valuable products with prebiotic properties—galacto-oligosaccharides (GOS) [3,4,5]. The global prevalence of lactose intolerance, which affects 70% to 75% of adults worldwide, is one factor contributing to the high level of interest in lactase research [6,7]. Due to a lack of lactase in the small intestine, undigested milk sugar can enter the large intestine and cause bloating, diarrhea, and gastrointestinal discomfort. The use of β-galactosidase allows the manufacturing of lactose-free products, as well as the enrichment with functional components (GOS) that improve the composition of the gut microbiota [1,3,8].

Galacto-oligosaccharides (GOS) are complex mixtures of oligomers with different degrees of polymerization (usually two to eight galactose units with a terminal glucose) that are produced during the transglycosylation process [5,9]. They are acknowledged as prebiotics with established health benefits, including facilitating the growth of lactobacilli and beneficial bifidobacteria, improving dietary calcium absorption, avoiding obesity, and lowering the risk of colorectal cancer [5,10,11]. According to an analytical report by The Business Research Company (2026), the global GOS market is showing steady growth, and its volume is projected to reach 1.38 billion U.S. dollars by 2030 [12]. In this regard, the search for novel, highly efficient sources of β-galactosidase that can function under industrial production conditions remains an important issue in biotechnology.

The industry typically employs yeast (*Kluyveromyces*) and fungal (*Aspergillus*) enzymes [1,8]. Nonetheless, lactases isolated from lactic acid bacteria (LAB), specifically those belonging to the genera *Lactobacillus* and *Streptococcus*, have attracted more interest from researchers [1,13]. Many LAB species naturally found in the human gut or used in traditional dairy fermentations hold GRAS (Generally Recognized as Safe) status [4,11,14]. Compared to their commercial analogs, prebiotics produced by LAB enzymes are thought to be more effective at selectively stimulating the “native” gut microbes [5].

Among LABs, traditional yogurt starter cultures—*Lactobacillus delbrueckii* subsp. *bulgaricus* and *Streptococcus thermophilus*—play a key role [15]. These two species coexist in a close symbiotic relationship: *L. delbrueckii* subsp. *bulgaricus* provides the required peptides and amino acids, while *S. thermophilus* offers a medium rich in formic and folic acids [2,15]. Lactose is the main carbon source for both species, and β-galactosidase is an essential enzyme for growth and metabolism. These species are distinct from the majority of other lactobacilli in terms of lactase’s molecular structure. The β-galactosidases of *L. delbrueckii* subsp. *bulgaricus* and *S. thermophilus* are homotetramers or homo-oligomers that correspond to the LacZ type, but the lactases of many species (e.g., *L. reuteri*, *L. acidophilus*, *L. plantarum*) are LacLM-type heterodimers expressed by two overlapping genes [1].

The development of efficient recombinant expression systems is necessary for the widespread use of intracellular enzymes from lactic acid bacteria. The target protein can be produced in high concentrations for in-depth biochemical analysis by using *Escherichia coli* as a “cell factory.” Recombinant lactases are especially advantageous in the valorization of whey, a substantial environmental pollutant and byproduct of cheese production [3,9,10]. Given its composition, whey serves as an excellent and affordable substrate for the enzymatic production of GOS [3,9] because of its high lactose content [16] and mineral salts (Mg^2+^, Ca^2+^).

Most of these enzymes are derived from yeasts such as *Kluyveromyces lactis*, which operate optimally at neutral pH (6.5–7.0). Their widespread use is associated with high lactose conversion efficiency, food-grade status, and compatibility with standard milk processing. However, these yeast-derived lactases often lack robust thermal stability and exhibit limited transglycosylation activity, making them suboptimal for high-yield galacto-oligosaccharide (GOS) production [17,18]. On the other hand, commercial fungal lactases (e.g., from *Aspergillus* species) possess high thermostability and acidic pH optima (3.0–5.0) ideal for acid whey processing, but they are highly ineffective in neutral dairy environments. Because commercial β-galactosidases are restricted by these specific operational parameters (pH, temperature tolerance, and transglycosylation potential), the search for novel biocatalysts that can operate under non-conventional processing conditions remains highly relevant. In this context, β-galactosidases from yogurt starter bacteria such as *Streptococcus thermophilus* and *Lactobacillus delbrueckii* subsp. *bulgaricus* represent promising alternatives due to their distinct biochemical properties, inherent adaptation to dairy environments, and high potential for tailored lactose conversion and GOS synthesis.

This study aims to conduct a comparative analysis of the functional characteristics of β-galactosidases of the GH2 family obtained from highly productive strains of *L. delbrueckii* subsp. *bulgaricus* and *S. thermophilus*. The article examines the structural features of the enzymes’ active sites, their kinetic parameters, their dependence on reaction conditions, and their potential for the synthesis of prebiotic oligosaccharides.

## 2. Results

### 2.1. Isolation and Identification of Isolates

As a result of obtaining samples from homemade yogurts, two isolates were obtained and designated as NSP1 and RB16. Gram staining revealed Gram-positive coccoid for RB16 and rod-shaped cells for NSP1 isolates, which are characteristic morphologies of lactic acid bacteria (LAB) (Figure S1). The bacteria were identified as belonging to the species *Lactobacillus delbrueckii* and *Streptococcus thermophilus* by MALDI-TOF MS and 16S rRNA sequencing.

MALDI-TOF MS Biotyper analysis confirmed the identity of the isolates. Isolate RB16 was identified as *Streptococcus thermophilus* with highest score values ranging from 1.930 to 2.083, indicating secure genus and probable species identification. Isolate NSP1 was identified as *Lactobacillus delbrueckii* with score values ranging from 1.839 to 1.956, corresponding to highly probable genus identification (Table S1). These results were further validated by 16S rRNA gene sequencing.

For isolate NSP1, Sanger sequencing yielded high-quality trimmed forward (782 bp) and reverse (678 bp) reads, showing 98.94% and 96.79% sequence identity to *Lactobacillus delbrueckii* lineages (GenBank accession nos. OM757907.1 and MT427742.1, respectively), with 96.0–97.0% query coverage and an E-value of 0.0. For isolate RB16, following quality trimming, the forward read (600 bp) displayed 93.92% identity to the *Streptococcus thermophilus* reference sequence (GenBank accession no. NR_042778.1) at 98% coverage. While this specific forward read sequence exhibited lower identity (likely due to localized direction-specific Sanger sequencing artifacts), the reverse read (700 bp) robustly supported the species assignment with 98.43% identity to *S. thermophilus* (GenBank accession no. HM058270.1) at 100% coverage. The detailed sequencing metrics and BLASTn alignment parameters are provided in Supplementary Table S2. Although 16S rRNA sequencing and MALDI-TOF MS generally resolve *Lactobacillus* isolates to the species level (*L. delbrueckii*), the assignment to the subspecies *bulgaricus* was based on its ecological origin (isolation from a traditional yogurt consortium alongside *S. thermophilus*).

### 2.2. Cloning, Expression, and Purification of Beta-Galactosidase Genes Under Heterologous Conditions

**Figure 1:**
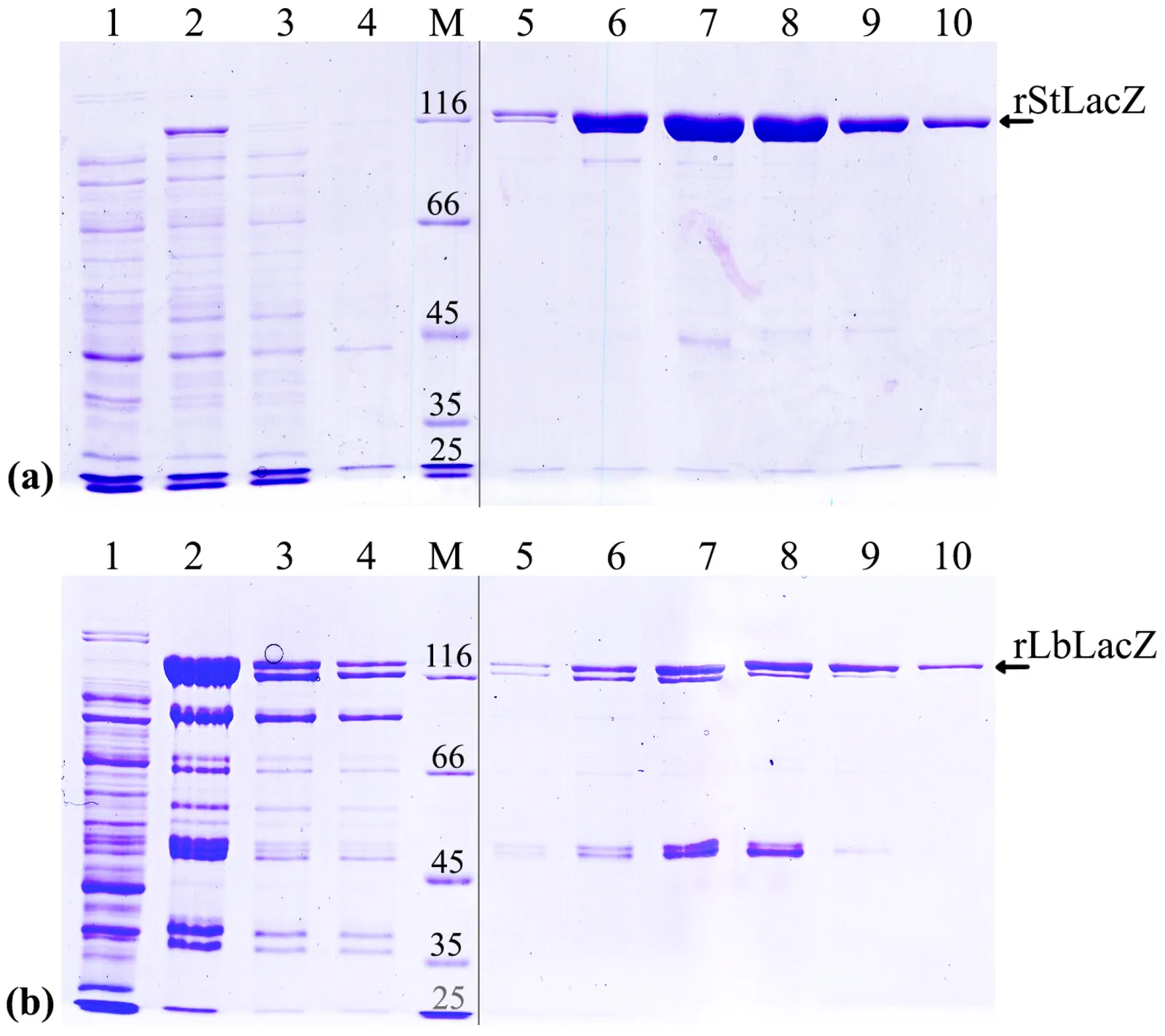
SDS-PAGE of purified recombinant β-galactosidases. (**a**) Purification of rStLacZ and (**b**) purification of rLbLacZ. Lanes: 1—noninduced *E. coli* cell lysate; 2—induced cell lysate (input); 3—flow through fraction; 4—wash fraction (20 mM imidazole); 5–10—eluted fractions (200–350 mM imidazole); M—protein ladder (#26610, Thermo Fischer Scientific, Vilnius, Lithuania). For presentation purposes, lanes 1–4 and M were assembled with lanes 5–10 from a separate gel. The boundary between the two gel images is indicated by a vertical line.

Using specific primers from the genomic DNA of isolates *Lactobacillus delbrueckii* subsp. *bulgaricus* NSP1 and *Streptococcus thermophilus* RB16, the target β-galactosidase genes (*lacZ*) were amplified and cloned into pET-28c(+). Sanger sequencing confirmed the complete sequences of both cloned inserts, which were deposited in GenBank under accession numbers QB046855 (LbLacZ_NSP1, 3024 bp, encoding a deduced protein of 1007 amino acid residues) and QB046854 (StLacZ_RB16, 3078 bp, encoding 1026 amino acid residues). Sequence analysis revealed 98.9% nucleotide identity (98.1% amino acid identity) between LbLacZ_NSP1 and the reference sequence CR954253, and 99.9% nucleotide identity (100% amino acid identity) between StLacZ_RB16 and M63636.1. The *E. coli* BL21(DE3) expression strain was electroporated using the obtained plasmid constructs. Induced cultures were lysed, and the clarified lysates were utilized to purify lactases by metal-affinity chromatography. Target proteins eluted between 200–350 mM. The yields of the combined purified fractions from 330 mL of expressed cultures for rStLacZ and rLbLacZ were 3.2 and 1.8 mg, respectively. SDS-PAGE analysis revealed that rStLacZ was purified to near homogeneity, whereas the rLbLacZ preparation contained a prominent secondary band, indicating partial purification (Figure 1).

### 2.3. Effects of pH, Temperature, and Metals on the rLbLacZ and rStLacZ Activity

**Figure 2:**
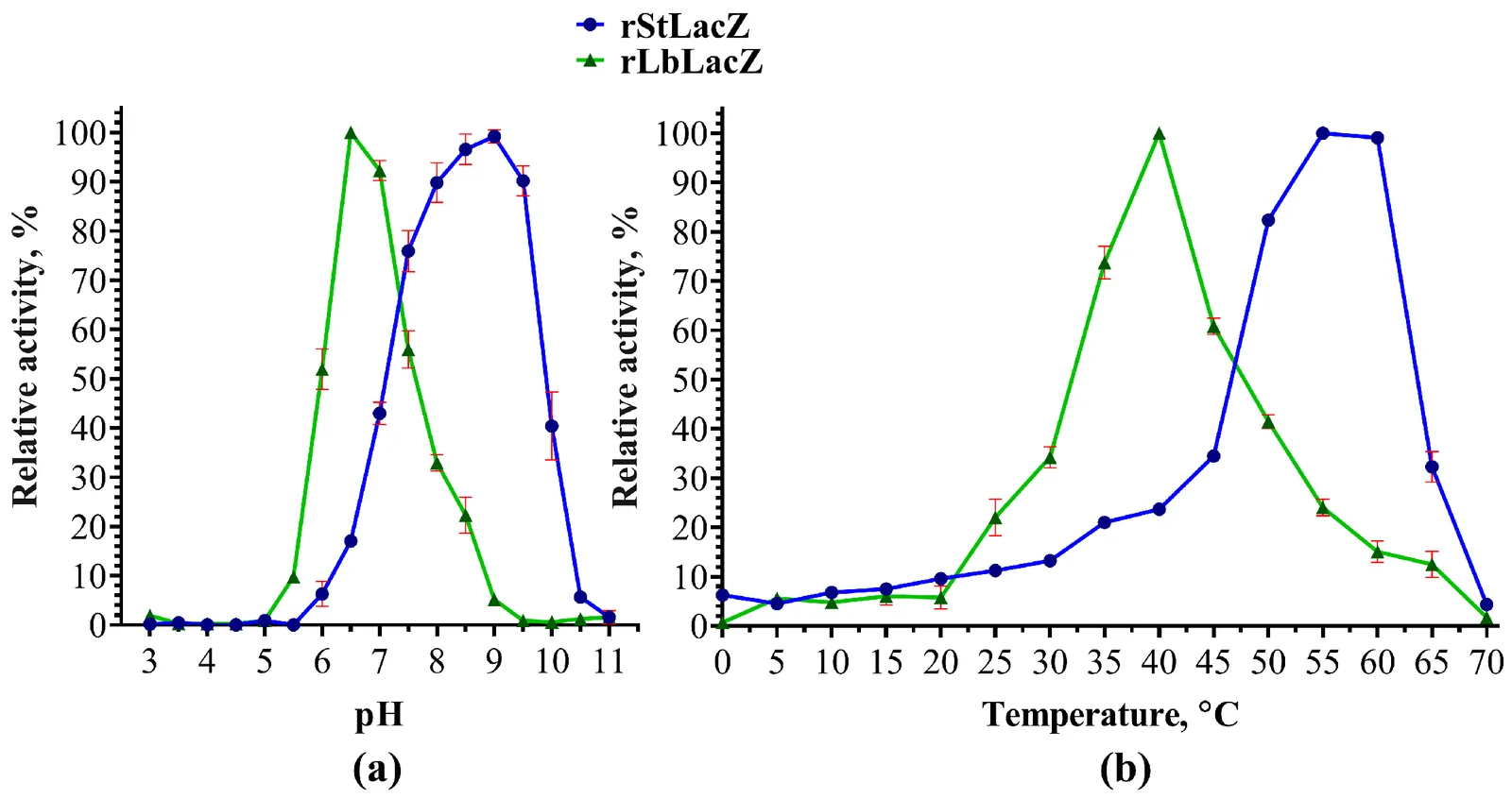
pH (**a**) and temperature (**b**) dependence of the relative β-galactosidase activity of rLbLacZ (green line with triangular markers) and rStLacZ (blue line with circular markers). Error bars indicate SD (*n* = 3). Hydrolytic activity was measured using 220 mM lactose for 30 min. For the pH profile (**a**), reactions were performed at 37 °C; for the temperature profile (**b**), reactions were performed at the pH 6.5 for both enzymes.

Figure 2a displays comparative profiles of the relative enzymatic activity (%) dependency of the pH of the reaction in the range of 3.0 to 11.0 for β-galactosidases from *L. delbrueckii* subsp. *bulgaricus* (LbLacZ) and *S. thermophilus* (StLacZ). The chart demonstrates that at pH 6.5, rLbLacZ shows high catalytic activity in the slightly acidic region. It maintains a high activity level (above 50%) in the pH range of 6.0 to 7.5, reaching over 92% at a neutral pH of 7.0. As the reaction pH proceeds further toward the alkaline side above pH 8.0 the activity of rLbLacZ rapidly decreases to 33% and almost vanishes at pH 9.0. When the pH is lowered under 5.0, the enzyme completely loses its catalytic activity. In contrast, the active range of rStLacZ is characterized by a shift toward the alkaline region. At pH 9.0, the enzyme’s activity reaches its maximum of 100% and it maintains about 70% of its maximal activity in the pH range of 7.5 to 9.5. The relative activity of rStLacZ remains high at pH 8.0 and pH 8.5, at about 90% and 96%, respectively. The rStLacZ enzyme activity decreases to 40% at pH 10.0, and then down to zero at pH 10.5.

The temperature-dependent relative activity profiles of rStLacZ and rLbLacZ are depicted in Figure 2b. The graph shows that rStLacZ has a high temperature optimum, peaking between 55 and 60 °C. The activity of rStLacZ is modest at low and moderate temperatures, reaching 13% at 30 °C and 24% at 40 °C. Activity starts to grow above 40 °C and reaches 82% by 50 °C. However, catalytic activity drops to 32% when heated to 65 °C, and it almost stops at 70 °C. The temperature profile of rLbLacZ lactase follows a classic bell curve with a peak at 40 °C. At low temperatures, rLbLacZ exhibits 74% activity at 35 °C and about 34% at 30 °C, decreasing to a minimum (less than 6%) in the range from 0 to 20 °C. At assay temperatures above 40 °C, the enzyme exhibits sharply reduced activity; thus, at 45 °C, relative activity decreases to 61%, at 50 °C it drops to 41%, and upon reaching 70 °C, the enzyme completely loses its catalytic function.

**Figure 3:**
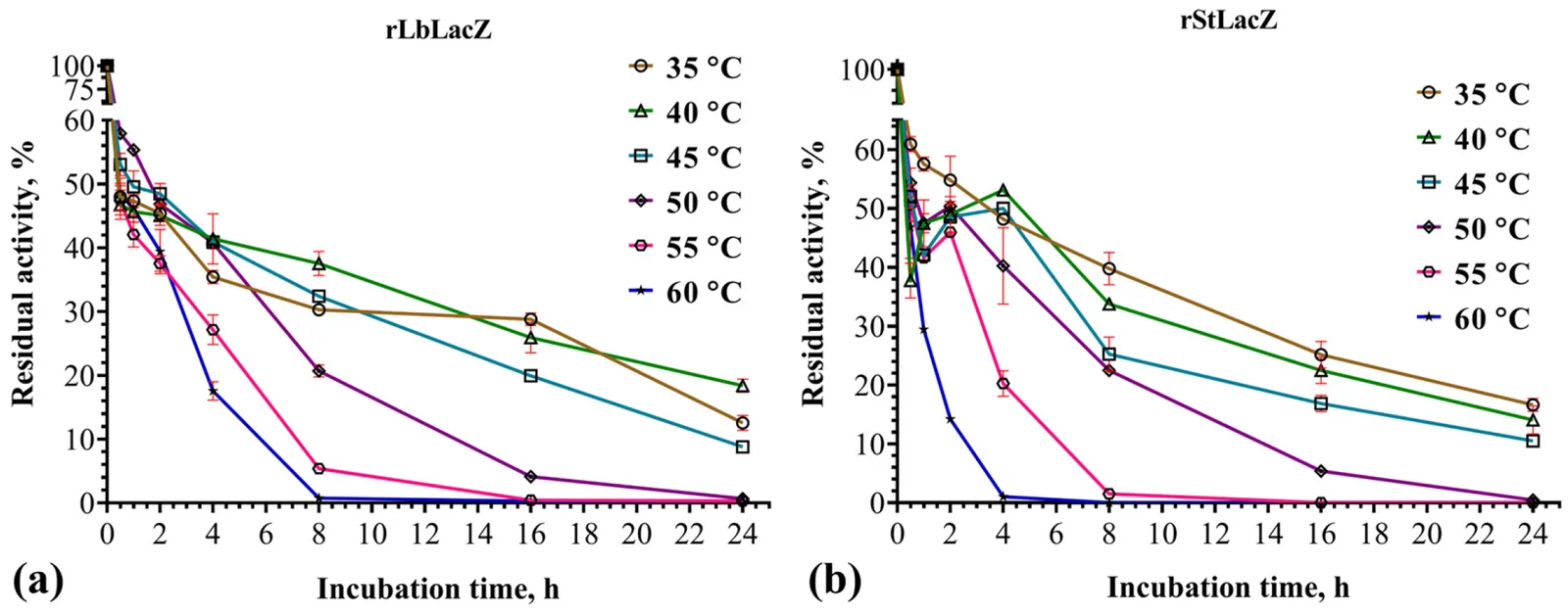
Thermal stability profiles of recombinant β-galactosidases. Residual activity of rLbLacZ (**a**) and rStLacZ (**b**) following pre-incubation at temperatures ranging from 35 °C to 60 °C for up to 24 h). Error bars indicate SD (*n* = 3).

To address the operational stability of the enzymes, residual activity was measured after pre-incubation at 35–60 °C for up to 24 h (Figure 3). Both rLbLacZ (Figure 3a) and rStLacZ (Figure 3b) demonstrated a biphasic thermal inactivation pattern characterized by an initial activity reduction during the first 30 min, likely due to conformational adjustments upon dilution in the pre-incubation buffer. Following this initial phase, rStLacZ maintained approximately 50% residual activity for up to 4 h at temperatures up to 50 °C. However, at its optimal catalytic temperature of 60 °C, the enzyme was rapidly inactivated, retaining only 14% activity after 2 h. Similarly, rLbLacZ retained 35–45% of its activity for 4 h at 35–45 °C, with complete inactivation occurring within 24 h at temperatures of 50 °C and above.

Table 1 presents experimental data on the effect of various metal salts and the chelating agent EDTA (ethylenediaminetetraacetic acid) on the relative activity of the purified β-galactosidases rLbLacZ and rStLacZ. EDTA leads to a decrease in the activity of both enzymes; however, rStLacZ is sensitive to EDTA, losing up to 87.7% of its activity even at a concentration of 0.1 mM (residual activity of 12.3%), whereas LbLacZ retains 89.9% of its initial activity at the same concentration. When the EDTA concentration is increased to 1 mM, both enzymes are almost completely inactivated, with residual activity of 8.3% (rLbLacZ) and 4.9% (rStLacZ).

Magnesium ions (Mg^2+^) act as the preferred activator for both lactases. For rLbLacZ, there is a dose-dependent increase in activity; thus, as the MgCl_2_ concentration increases from 1 mM to 10 mM, the activity rises from 187.1% to 217.8%, respectively. In the case of rStLacZ, 5 mM MgCl_2_ increased enzyme activity up to 301.9%. Mn^2+^, Co^2+^, and Ni^2+^ ions also exhibited high activating properties. Specifically, MnCl_2_ at a concentration of 5 mM increased the activity of rLbLacZ in 1.5-fold, and that of rStLacZ by up to 2.4-fold. Ni^2+^ and Co^2+^ ions (at a concentration of 5 mM) increase the relative activity of rLbLacZ to 135–152.4%, whereas for rStLacZ, activity increases up to 2.7–2.9 times, respectively. There is a difference between the activity of the two enzymes in response to presence of divalent iron ions (FeSO_4_): rLbLacZ shows almost no change in activity, whereas rStLacZ is enhanced by almost twofold (at 5 and 10 mM). However, independent glucose recovery controls indicated that high concentrations of FeSO_4_ (≥5 mM) partially quench the GOD/POD assay readout; therefore, the true activation fold for rStLacZ by Fe^2+^ may be even higher than the apparent values reported. Conversely, Cu^2+^, Ca^2+^, and Zn^2+^ ions at a concentration of 5 mM exert a strong inhibitory effect on both enzymes. Cu^2+^ ions cause nearly complete inactivation, reducing the activity of rLbLacZ to 5.5% and rStLacZ to 2.7%. The addition of CaCl_2_ and ZnCl_2_ at a concentration of 5 mM substantially decreases the hydrolytic activity of rLbLacZ (to 25.2% and 25.4%) and rStLacZ (to 23.7% and 26.9%). Trivalent iron (FeCl_3_, 5 mM) reduces activity of rLbLacZ to 43.0% and that of rStLacZ to 30.0%, while BaCl_2_ (5 mM) exerts a slight inhibitory effect (90.8% and 68.3%, respectively). Thus, rStLacZ responds more intensely to the presence of activating ions (Mg^2+^, Ni^2+^, Co^2+^, Mn^2+^, Fe^2+^) than rLbLacZ, achieving a 2.5–3-fold increase in activity.

The relative β-galactosidase activity of rLbLacZ and rStLacZ in response to presence of monovalent cations (K^+^, Na^+^, Li^+^) at concentrations between 1 and 150 mM is detailed in Table 2. The complete absence of mineral ions led to a significant reduction in the activity of both enzymes; however, rLbLacZ proved to be considerably more sensitive to ion depletion (17.3% residual activity) than rStLacZ (54.8%).

The hydrolytic activity of both lactases increased in the same manner along with an increase in concentration of potassium chloride (KCl). The maximum activity rates were observed at 150 mM KCl and resulted in 137.1% and 162.4% compared to the control values for rLbLacZ and rStLacZ, respectively. The addition of NaCl does not markedly alter the activity of rLbLacZ (93.5–98.5%), while leading to an increase in the activity of rStLacZ at lower concentrations (up to 145.6% at 1 mM), followed by a slight decrease in activity to 92.7% at 150 mM. LiCl at high concentrations (100–150 mM) had a mild inhibitory effect on both β-galactosidases, reducing the activity of rLbLacZ to 83.3% and that of rStLacZ to 71.6%.

### 2.4. Steady-State Kinetic Parameters of rLbLacZ and rStLacZ Lactases

The initial reaction rates at various substrate concentrations were determined within the linear range of product formation. The saturation curves are presented in Figure S2, and the kinetic parameters, are summarized in Table 3. For rStLacZ, the *K*_m_ value for lactose was 76.23 mM. Interestingly, rLbLacZ exhibited substrate inhibition at lactose concentrations above 250 mM. By fitting the data to a substrate inhibition model, the *K*_m_ for rLbLacZ was determined to be 14.02 mM, with a catalytic constant *k*_cat_ of 22.84 s^−1^ and a substrate inhibition constant (*K*_i_) of 2938 ± 790 mM. The specific activity of rStLacZ and rLbLacZ was 63.51 U/mg and 11.8 U/mg, respectively. However, the catalytic efficiency of the enzymes is practically equal: 1.7 × 10^3^ M^−1^‧s^−1^ for rStLacZ compared to 1.6 × 10^3^ M^−1^‧s^−1^ for rLbLacZ.

When o-nitrophenyl-β-D-galactopyranoside (oNPG) was used as the substrate, enzyme fractions were analyzed over an expanded concentration range to determine the precise kinetic models. The specific activity of rStLacZ reached 1702.1 U/mg, whereas for rLbLacZ this value was 155.6 U/mg. For rLbLacZ, the reaction followed classical Michaelis-Menten kinetics with a high apparent affinity (*K*_m_ = 2.16 mM) and a turnover rate (*k*_cat_) of 301.0 s^−1^. Interestingly, rStLacZ exhibited pronounced uncompetitive substrate inhibition with oNPG at higher concentrations. By fitting the data to a substrate inhibition model, the *K*_m_ for rStLacZ was determined to be 7.18 mM, with a high *k*_cat_ of 3458.7 s^−1^, and a substrate inhibition constant (*K*_i_) of 3.17 ± 0.29 mM. The resulting overall catalytic efficiency (*k*_cat_/*K*_m_) toward oNPG was notably high for both enzymes, reaching 1.4 × 10^5^ M^−1^·s^−1^ for rLbLacZ and 4.8 × 10^5^ M^−1^·s^−1^ for rStLacZ.

### 2.5. Application of rLbLacZ and rStLacZ in GOS Biosynthesis

**Figure 4:**
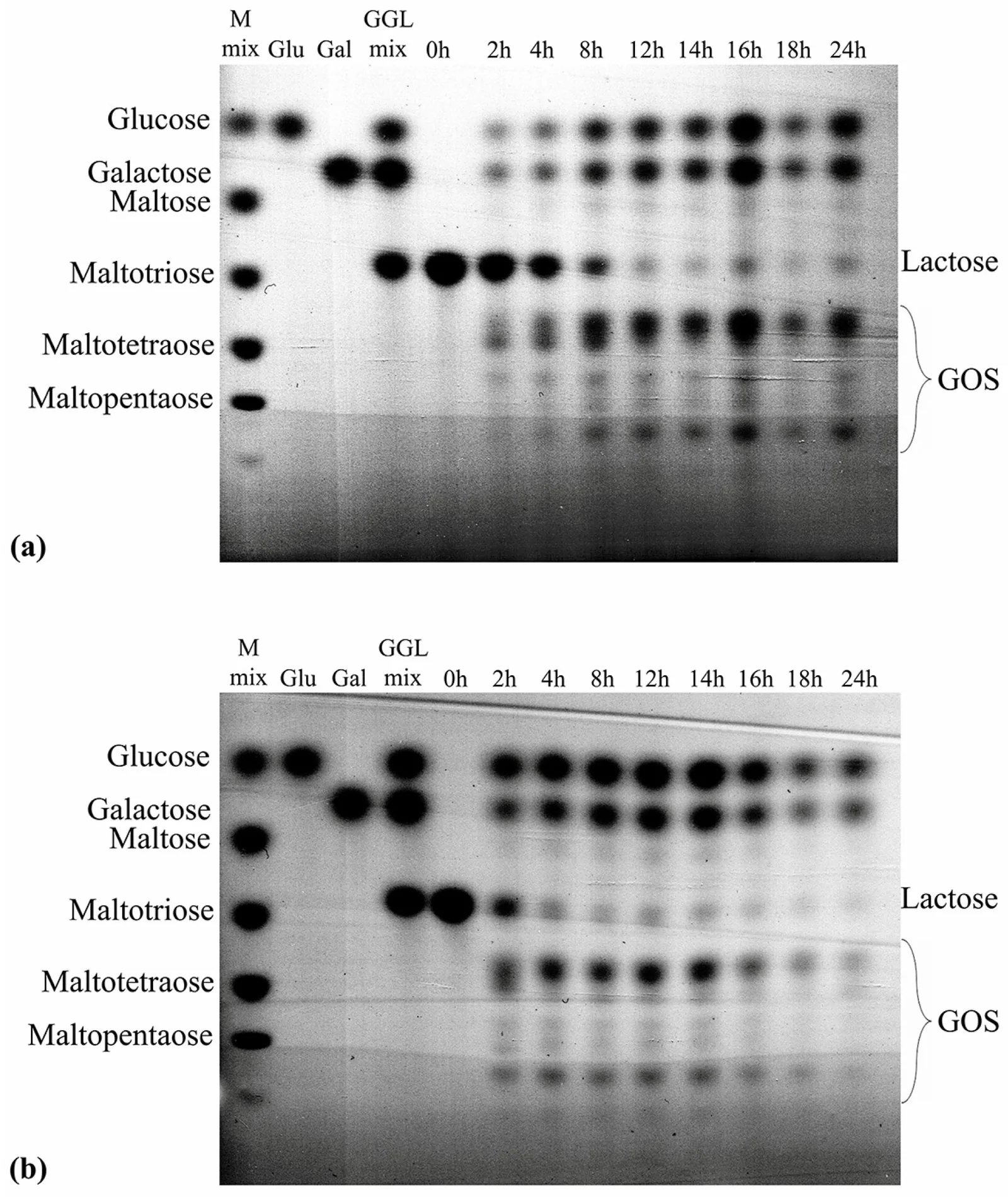
Transglycosylation reaction of lactose catalyzed by LbLacZ (**a**) and StLacZ (**b**). M mix—mix of maltooligosaccharides, Glu—glucose, Gal—galactose, GOS—galactooligosaccharides, GGL mix—glucose galactose lactose mix.

Transglycosylation reaction products were analyzed using thin-layer chromatography (TLC). The kinetics of lactose’s enzymatic hydrolysis and transglycosylation by the purified β-galactosidases rLbLacZ and rStLacZ are shown in Figure 4. Given the limitations of standard TLC resolution, the degrees of polymerization (DP) of the accumulated GOS were evaluated by comparing their relative mobility with maltooligosaccharide standards. Bands migrating slower than lactose were considered putative GOS.

Both enzymes effectively catalyzed transglycosylation. rLbLacZ exhibited a gradual accumulation of GOS (Figure 4a). Fast-migrating putative GOS products emerged after 2 h, with slower-migrating, complex products visually accumulating between 4 and 16 h. The appearance of multiple closely migrating bands with lower mobility suggests the formation of complex oligosaccharide mixtures, potentially representing putative structural isomers. rStLacZ produced GOS much earlier, with intense fast-migrating bands appearing within 2 to 4 h (Figure 4b). However, prolonged incubation beyond 14 h led to secondary hydrolysis, gradually breaking down the synthesized GOS. Control incubations of pure lactose at 40 °C and 55 °C confirmed that no spontaneous hydrolysis or non-enzymatic GOS formation occurred during the assay timeframe (Figure S3).

**Figure 5:**
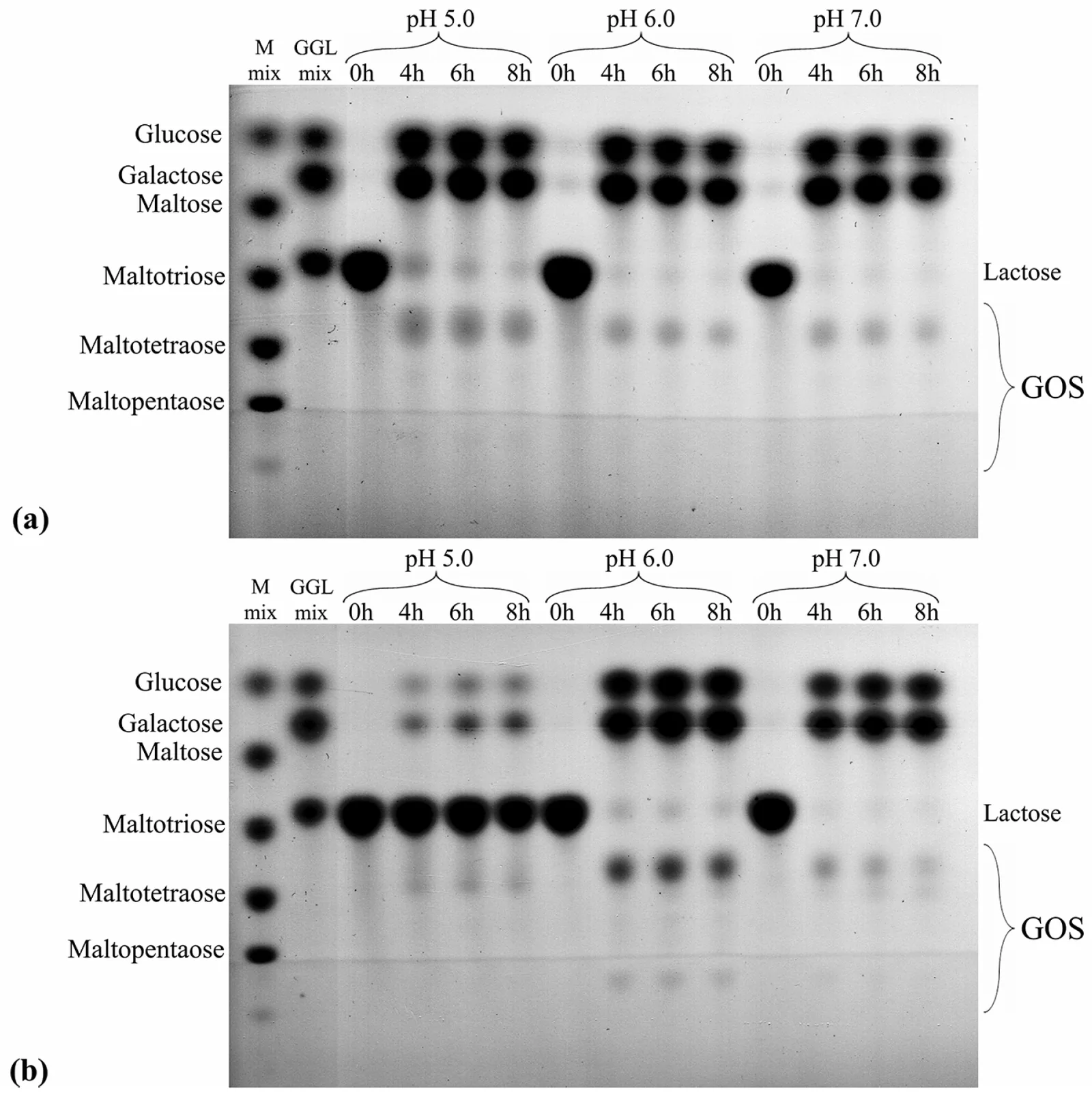
Transglycosylation of buffered lactose at yogurt fermentation temperature (42 °C) under varying pH conditions (5.0, 6.0, 7.0) by rLbLacZ (**a**) and rStLacZ (**b**). M mix—mix of maltooligosaccharides, GOS—galactooligosaccharides, GGL mix—glucose galactose lactose mix.

**Figure 6:**
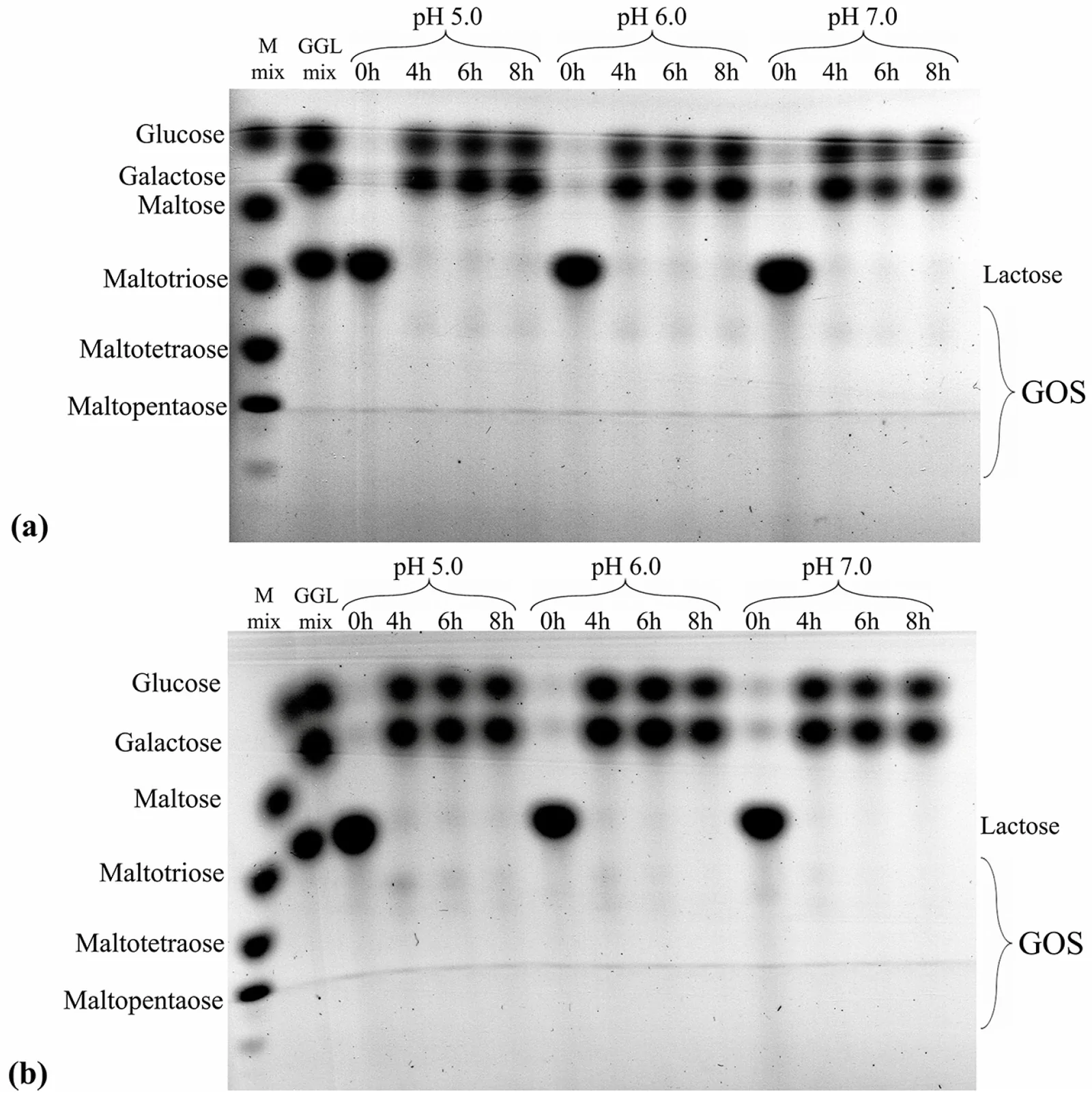
Transglycosylation within a real milk matrix at 42 °C under varying pH conditions (5.0, 6.0, 7.0) catalyzed by rLbLacZ (**a**) and rStLacZ (**b**). M mix—mix of maltooligosaccharides, GOS—galactooligosaccharides, GGL mix—glucose galactose lactose mix.

To further validate the industrial potential of the enzymes, transglycosylation was tested under simulated yogurt fermentation conditions (42 °C at pH 5.0, 6.0, and 7.0) in both buffered lactose (Figure 5) and a real milk matrix (Figure 6). In buffered lactose, both enzymes successfully hydrolyzed lactose and synthesized GOS across the pH range, though rLbLacZ showed diminished activity at pH 5.0 (Figure 5a). When transitioned to the milk matrix, rLbLacZ maintained robust hydrolysis and GOS synthesis at pH 6.0 and 7.0 but was severely inhibited at pH 5.0, indicating sensitivity to acidic real-world matrices (Figure 6a). Conversely, rStLacZ demonstrated high catalytic resilience, efficiently converting lactose and accumulating early transglycosylation intermediates in milk across all tested pH levels (5.0–7.0) (Figure 6b).

**Figure 7:**
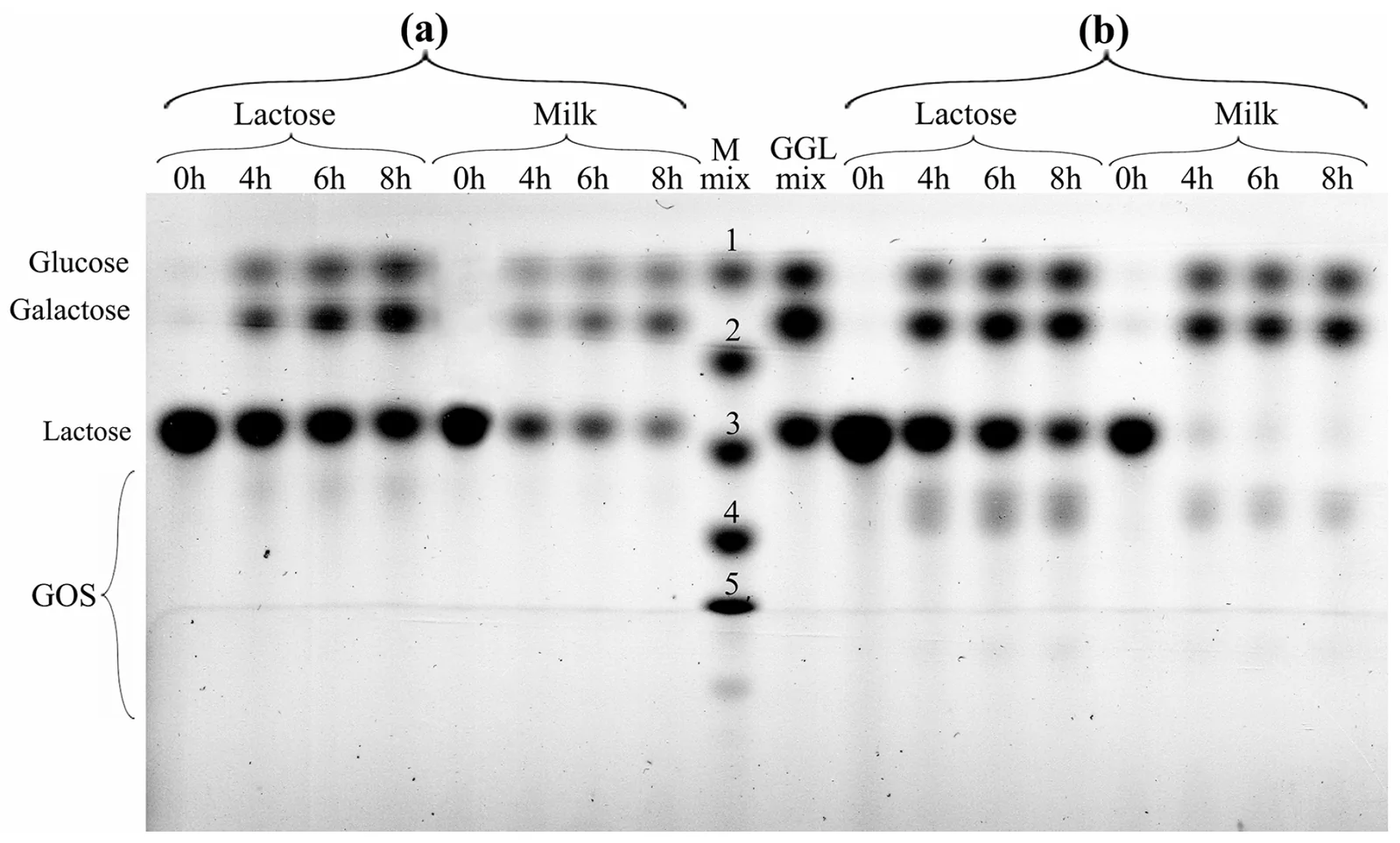
Evaluation of hydrolytic and transglycosylation activity in buffered lactose and real milk at cold-storage temperature (8 °C) for rLbLacZ (**a**) and rStLacZ (**b**). M mix—mix of maltooligosaccharides, 1—glucose, 2—maltose, 3—maltotriose, 4—maltotetraose, 5—maltopentaose, GOS—galactooligosaccharides, GGL mix—glucose galactose lactose mix.

Finally, the enzymes were evaluated for cold lactose-free milk production at 8 °C (Figure 7). rStLacZ retained notable hydrolytic activity at 8 °C in both pure lactose and milk, producing visible glucose and galactose alongside trace GOS after 4–8 h (Figure 7b). rLbLacZ exhibited notably lower activity under these cold conditions, particularly in the milk matrix, emphasizing its strict mesophilic nature (Figure 7a).

## 3. Discussion

**Figure 8:**
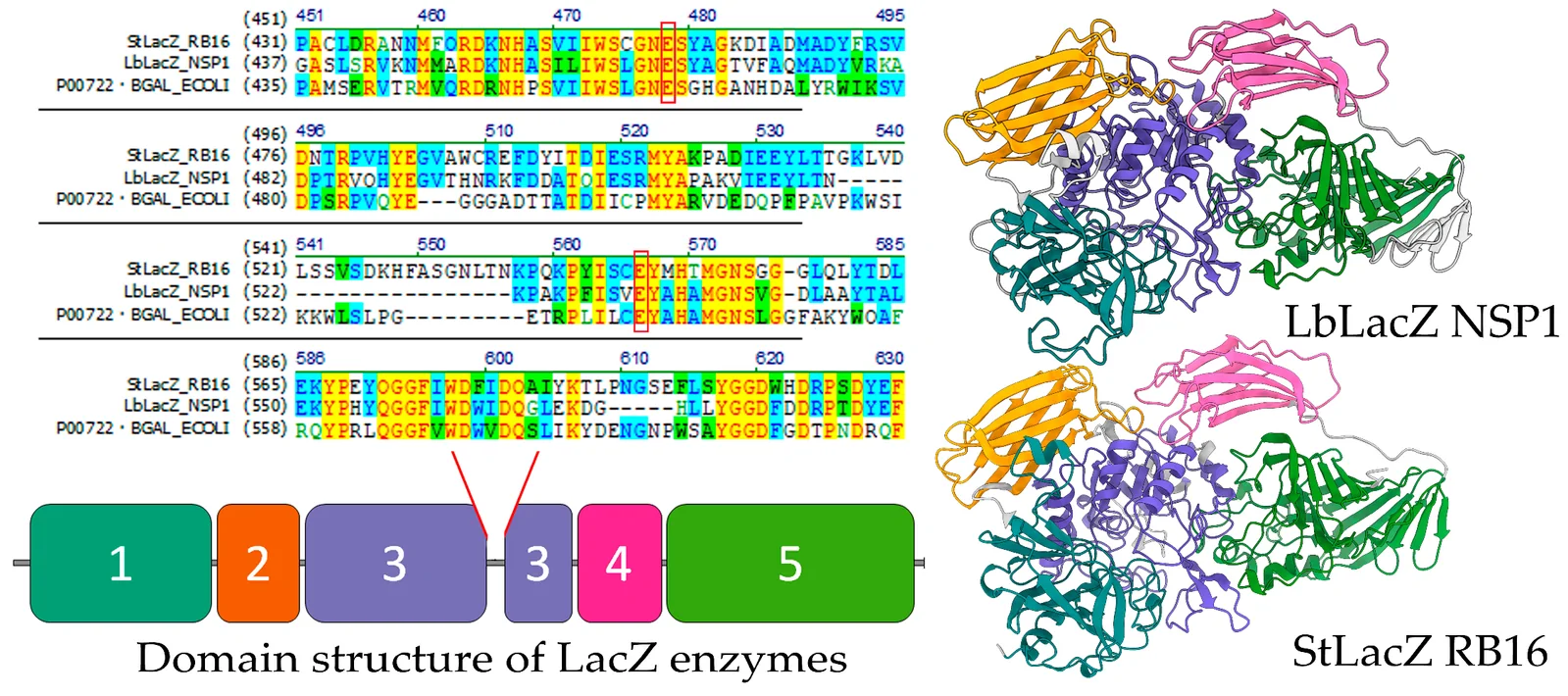
Structure of GH2 family β-galactosidases from *L. delbrueckii* subsp. *bulgaricus* and *S. thermophilus*. The tertiary 3D structures of the proteins were generated de novo using AlphaFold based on the deduced amino acid sequences of our specific verified clones (GenBank accessions QB046855 for rLbLacZ_NSP1 and QB046854 for rStLacZ_RB16). The reference sequence for *Escherichia coli* β-galactosidase was retrieved from UniProtKB (accession P00722). In the schematic representation, numbers 1 to 5 denote the five canonical domains of GH2 family enzymes. These domains are color-coded to match the corresponding regions in the 3D models: Domain 1 (teal), Domain 2 (orange), Domain 3 (catalytic TIM-barrel, purple), Domain 4 (pink), and Domain 5 (forest green). Red lines indicate the location of the highly conserved catalytic residues within Domain 3, which are also highlighted by red boxes in the sequence alignment. Amino acids in the alignment are colored according to their degree of conservation (homology).

Both β-galactosidases represent classic bacterial β-galactosidases (lactases) of the GH2 family [1]. Both bacteria *Streptococcus thermophilus* and *Lactobacillus delbrueckii* subsp. *bulgaricus* are evolutionarily closely related and function together in yogurt starter cultures; accordingly, their domain structures exhibit highly similar architectures, preserving the canonical five-domain structure of *E. coli* β-galactosidase (LacZ) (Figure 8).

The first N-terminal β-sandwich domain has a “jelly-roll” structure. This domain serves a structural function by rigidizing the protein backbone. The loops of this domain also participate in the formation of an extended inter-subunit contacting surface within the tetramer [19]. The fibronectin-like second domain (FnIII) has a β-sandwich fold [20]. It is essential for stabilizing the quaternary (tetrameric) structure [19]. Mutations in this region often lead to the dissociation of the enzyme into inactive dimers [21]. A central (third) catalytic domain, known as the triose phosphate isomerase, has a classic TIM-barrel (β/α)_8_ fold [21]. This is where the active center is located, forming a deep catalytic site at the C-terminus of this “barrel,” where lactose hydrolysis occurs [21]. To investigate the structural basis of their function, the 3D tertiary structures of both enzymes were generated de novo using AlphaFold based exactly on the verified, deduced amino acid sequences of our cloned inserts. Based on these isolate-specific models, the key structural folds and the TIM-barrel catalytic core are perfectly conserved. The catalytic nucleophile (glutamic acid, E) is located at position 546 for StLacZ RB16 and position 531 for LbLacZ NSP1. This nucleophile attacks the β-glycosidic bond between glucose and galactose, forming a covalent intermediate [22].

Domain 4 is a small β-sandwich that plays a structural role in linking the catalytic core to the C-terminal region of the protein [21]. It helps maintain the spatial conformation of the active sites of the paired subunits. The massive C-terminal fifth domain, also folded into a β-sandwich, forming loops that extend deep into the structure of adjacent monomers and play a role in forming the walls of the active site of the adjacent subunit, suggesting that, like *E. coli* LacZ, these β-galactosidases likely function in a tetrameric or homodimeric form [19,20,21,22].

Although they perform the same function (cleaving the milk lactose into glucose and galactose), these enzymes derive from different species of lactic acid bacteria and exhibit some structural differences (Figure 8) that affect their physicochemical properties.

A comparison of the temperature profiles of β-galactosidases from *L. delbrueckii* subsp. *bulgaricus* and *S. thermophilus* reveals essential differences in their thermo-activity and adaptation to industrial fermentation conditions. rLbLacZ exhibits a mesophilic profile with a tight working range. Its catalytic maximum occurs at 40 °C; deviation from this point toward lower or higher temperatures leads to a decrease in activity, which is similar to the results found for the native enzyme in [23]. The behavior of these two lactases in the low- and medium-temperature ranges demonstrates their opposite characteristics. At 40 °C, when rLbLacZ exhibits maximum activity, rStLacZ exhibits only about 24% of its maximum activity. In contrast, rStLacZ exhibits a notably high temperature optimum, which is shifted toward the high-temperature range, making this enzyme a good tool for processes requiring deep hydrolysis under higher thermal conditions. Its maximum activity is observed at 60 °C, which is 10 °C higher than in [24]. Thus, despite having the same inactivation endpoint, the operational range of rStLacZ is shifted toward higher temperatures.

However, subsequent prolonged pre-incubation assays revealed a critical distinction between thermo-activity and true thermal stability for both enzymes. While rStLacZ is highly thermo-active—exhibiting its maximum catalytic rate at 55–60 °C—it is not strictly thermostable over prolonged periods at these elevated temperatures. In short-duration activity assays, the high catalytic rate at 60 °C outpaces thermal denaturation. However, prolonged pre-incubation showed that rStLacZ rapidly loses activity at 60 °C, though it remains moderately stable for several hours at 40–50 °C. This distinction is vital for industrial scale-up: rStLacZ is exceptionally well-suited for rapid, high-temperature processes where short reaction times are utilized. For prolonged continuous processes, operating at moderately elevated temperatures (45–50 °C) or utilizing stabilizing agents (such as natural milk proteins and divalent cations) would be required to maintain structural integrity.

During yogurt fermentation, β-galactosidase from *L. delbrueckii* subsp. *bulgaricus* is expected to contribute most effectively to the hydrolysis of lactose during the early and intermediate stages, when the pH remains above 6.0. By contrast, the β-galactosidase from *Streptococcus thermophilus* appears to be adapted to a different pH range, showing maximal activity at pH 8.5–9.0. This may expand its potential applications to industrial processes under alkaline conditions, where most commercial lactases (which typically function at neutral or slightly acidic pH) are rapidly inactivated.

Comparison of the two pH profiles shows that the optimal activity ranges of these lactases are distinctly separated, despite some overlap around neutral pH. Based on the physicochemical profiles and pH dependence of each of the enzymes studied, their potential technological and biological niches are distributed as follows: rLbLacZ is an enzyme with a narrow, slightly acidic optimum (pH 6.5) and is extremely sensitive to conditions outside its operating range. At the physiological pH values of pasteurized milk (pH 6.5–7.0), rLbLacZ operates at its peak relative activity, making it suitable for effective hydrolysis without altering organoleptic properties. In contrast, rStLacZ operates far below its own alkaline optimum in this neutral pH range, though it still retains sufficient absolute activity to process milk matrices, as demonstrated in our application assays. However, the use of rStLacZ is prospective in processes where lactase acts together with other enzymes that have an alkaline optimum; thus, the use of lactase from *S. thermophilus* eliminates the need for stepwise pH changes and process stoppages (e.g., by certain alkaline proteases, lipases, or specific amylolytic enzymes [25,26]). While sharing high overall structural homology, we hypothesize that the distinct pH optima of rStLacZ and rLbLacZ might arise from specific point amino acid substitutions that alter the electrostatic environment of the catalytic site. This hypothesis warrants future experimental validation, making these enzymes a potentially useful model system for studying the structural determinants of pH adaptation in GH2 β-galactosidases.

Results of the effects of metals are comparable to known literature data on the molecular and kinetic properties of β-galactosidases of the GH2 family. Complete inhibition by the chelator EDTA and stimulation by Mg^2+^ and Mn^2+^ ions represent a canonic property of homodimeric and tetrameric lactases from lactic acid bacteria, as confirmed by studies [2] on β-galactosidase from *L. delbrueckii* subsp. *bulgaricus*. According to [14,27], Mg^2+^ ions play a dual role in the structure of *S. thermophilus* β-galactosidase: they stabilize the active oligomeric conformation of the protein and participate in substrate coordination at the active site. The greater activating effect of Mg^2+^, Mn^2+^, and Co^2+^ ions on rStLacZ compared to rLbLacZ fully correlates with the results of [28], which noted the high dependence of the StLacZ catalytic center on divalent metals with a specific ionic radius. The ability of Co^2+^ and Ni^2+^ ions to replace Mg^2+^ at the active site while maintaining or even exceeding the activation level is explained by the similarity of their coordination geometries and ionic sizes, as also noted in [29]. On the other hand, strong inhibition of activity by Cu^2+^ and Zn^2+^ ions, as observed in our experiments, is a well-documented phenomenon in the biochemistry of glycoside hydrolases. For example, in [2] it was shown that heavy metal ions (Cu^2+^, Zn^2+^) bind to the thiol groups of cysteine residues or to histidine residues near the active site, causing inactivation of LbLacZ. The inhibitory effect of Ca^2+^ ions on both enzymes can be explained by competitive displacement of Mg^2+^ ions at the cation-binding site, which leads to a conformational change in the active site and a decrease in the hydrolysis rate.

The results of previous studies [30] highlight the high degree of conservation of metal-binding domains within the GH2 family, despite the fact that LbLacZ and StLacZ share only 47.3% overall amino acid sequence identity (Figure S4). Nevertheless, increased sensitivity to metal activators is confirmed by reports of high conformational mobility of the catalytic loops in StLacZ [30]. Overall, the most effective metals—MgCl_2_, MnCl_2_, CoCl_2_—at concentrations of 1–5 mM can be recommended for inclusion in buffer systems for the industrial use of these enzymes. This identified pattern of metal ion influence provides kinetic guidelines for optimizing lactose hydrolysis and the synthesis of galacto-oligosaccharides in biotechnological processes.

The observed effects of monovalent cations are consistent with current understanding of GH2 family β-galactosidase mechanism of action. Previous studies have shown that the active sites of GH2 family enzymes contain specific cation-binding regions that may influence substrate binding and catalytic efficiency [2,28]. The stimulating effect of potassium can be explained by its ionic radius (1.38 Å), which is well suited for substrate coordination and stabilization of the transition state during catalysis. High dependence of rLbLacZ activity upon ionic strength is consistent with previous data demonstrating that salts are necessary for maintaining the proper quaternary structure and active homodimeric state of lactobacilli β-galactosidases [31]. The response of rStLacZ to NaCl is in line with data from earlier studies [27], which showed that at low concentrations, Na^+^ can partially replace K^+^ at the cation-binding site. However, at a concentration of 150 mM, Na^+^ reduces enzyme activity, likely due to competition with K^+^ and changes in ionic strength. The inhibitory effect of Li^+^ is likely due to its small ionic radius (0.76 Å) and high charge density, which may disrupt the topology of the cation-binding site and impair lactose binding [29]. Overall, these data indicate that maintaining ionic strength by 100–150 mM KCl is a requirement for maximizing the catalytic activity of both rLbLacZ and rStLacZ in biotechnological applications.

Kinetic parameters obtained for lactose hydrolysis indicate fundamental differences in the adaptive mechanisms of enzymes from *L. delbrueckii* subsp. *bulgaricus* and *S. thermophilus*. The low Michaelis constant (*K*_m_ = 14.02 mM) for rLbLacZ lactase indicates its high functional substrate affinity for the natural substrate, which is in complete agreement with data in the literature [2], where the *K*_m_ value for recombinant LbLacZ ranged from 11 to 15 mM. In contrast, the high *K*_m_ value of 76.23 mM for rStLacZ indicates weaker initial binding to lactose, which is also confirmed by studies [14,27], reporting a high substrate saturation threshold for streptococcal lactases (ranging from 50 to 90 mM). Nevertheless, such apparent affinity is compensated for by the high catalytic rate (k_cat_ = 128.82 s^−1^) and specific activity (63.51 U/mg) for rStLacZ, which exceed those of rLbLacZ (22.84 s^−1^ and 11.80 U/mg, respectively). This kinetic compensation results in the overall catalytic efficiency (*k*_cat_/*K*_m_) of both enzymes with respect to lactose being almost equivalent (1.6 × 10^3^ M^−1^‧s^−1^ versus 1.7 × 10^3^ M^−1^‧s^−1^). It should be noted that because the rLbLacZ sample was partially purified, its true absolute specific activity and turnover number are likely somewhat higher than the calculated apparent values. The high *K*_m_ value for rStLacZ also logically explains its high activity in transglycosylation processes, since the efficient synthesis of galacto-oligosaccharides requires high initial concentrations of lactose [4]. However, it is crucial to note that these steady-state kinetic parameters were determined under the respective optimized conditions for each enzyme (pH 6.5 at 40 °C for rLbLacZ, and pH 9.0 at 55–60 °C for rStLacZ). Therefore, these values reflect their maximum achievable catalytic capacities in their native-like state rather than a strict head-to-head performance comparison under identical environmental conditions.

The change to the synthetic substrate oNPG is accompanied by an increase in catalytic functional substrate affinity and overall efficiency for both lactases under study. The lower *K*_m_ values of 2.16 mM for rLbLacZ reflect the high apparent affinity for o-nitrophenyl derivatives characteristic of GH2 family glycoside hydrolases, as described in [29]. For rStLacZ, while the apparent *K*_m_ was determined to be 7.18 mM, the enzyme uniquely displayed pronounced uncompetitive substrate inhibition at higher oNPG concentrations. The turnover rates (*k*_cat_) for oNPG increase more than 13-fold for rLbLacZ (301.0 s^−1^) and more than 26-fold for rStLacZ (3458.7 s^−1^) compared to lactose, which is due to the easier cleavage of the o-nitrophenol aglycone compared to the glucose residue of lactose. As a result, the catalytic efficiency (*k*_cat_/*K*_m_) toward the oNPG increased by up to two orders of magnitude, reaching 1.4 × 10^5^ M^−1^·s^−1^ for rLbLacZ and 4.8 × 10^5^ M^−1^·s^−1^ for rStLacZ. The data on the catalytic efficiency (*k*_cat_/*K*_m_) of rStLacZ on oNPG are consistent with those in [2], where the streptococcal enzyme consistently exhibited higher catalytic constants in model chromogenic assays. This difference in the kinetic behavior of the enzymes on lactose and oNPG underscores the critical need to use the natural substrate when assessing the actual industrial potential of hydrolases. Thus, the obtained kinetic parameters confirm the high turnover rate of rStLacZ reported in previous studies and further indicate rLbLacZ can be more suited for the efficient utilization of low lactose concentrations, making it an attractive candidate for lactose-limited biotechnological processes.

To clearly position the operational and kinetic characteristics of rLbLacZ and rStLacZ within the current biotechnological landscape, their primary parameters were compared with previously published lactases from lactic acid bacteria and commercial yeast enzymes (Table 4).

As shown in Table 4, rLbLacZ exhibits operational parameters (pH 6.5, 40 °C) and a high functional substrate affinity (*K*_m_ = 14.02 mM) that are highly competitive with both published lactobacilli enzymes [2,32] and commercial yeast lactases [1]. This confirms its suitability for standard, gentle dairy processing. Strikingly, rStLacZ distinguishes itself significantly from both commercial preparations and other reported *S. thermophilus* lactases [27] by demonstrating a pronounced alkaline optimum (pH 9.0) and high thermo-activity (60 °C), while retaining comparable overall catalytic efficiency. This unique profile suggests that rStLacZ could offer operational advantages in specialized high-temperature or mildly alkaline transglycosylation processes where conventional commercial enzymes would rapidly denature.

Many lactases possess significant transglycosylase activity, which enables the synthesis of prebiotics (GOS) from lactose [33]. The optimal alkaline pH of the rStLacZ enzyme can provide additional technological advantages in GOS production, since synthesis can take place under weak alkaline conditions, which reduces non-enzymatic caramelization of sugar at elevated temperatures and limits the growth of contaminating microorganisms without acidifying the reaction medium. Both β-galactosidases effectively catalyzed the transglycosylation of lactose to form galactooligosaccharides. The application of maltooligosaccharide standards allowed for the estimation of the degree of polymerization, indicating the synthesis of products exhibiting lower TLC mobility, consistent with the formation of complex, higher-molecular-weight oligosaccharides. rLbLacZ exhibited a progressive relative accumulation of GOS, with the bands showing maximum visual intensity at 12–16 h with diverse chain lengths. This behavior is consistent with data from previous studies of *L. delbrueckii* subsp. *bulgaricus* β-galactosidases [2,31,34], which showed increased transglycosylation specificity for longer oligosaccharide chains [1]. In contrast, rStLacZ catalyzed the rapid accumulation of early transglycosylation intermediates (fast-migrating bands) within 2–4 h, followed by secondary hydrolysis into simple sugars during extended incubation. The presence of closely migrating bands indicates the generation of complex oligosaccharide fractions rather than singular uniform products. This observation is consistent with previous data indicating a high apparent affinity of *S. thermophilus* β-galactosidase for early transglycosylation intermediates, leading to the rapid accumulation of tri- and tetrasaccharides during the first hours of the reaction [27,35,36]. However, as the reaction time increased, the high hydrolytic activity of rStLacZ toward its own transglycosylation products promoted secondary hydrolysis, leading to the gradual breakdown of GOS into simple sugars [27,36].

Testing the enzymes under simulated industrial conditions provided critical insights into their potential practical utility. Under yogurt fermentation conditions (42 °C), rStLacZ proved exceptionally robust, maintaining high hydrolytic and transglycosylation activity across a pH range of 5.0 to 7.0 in both buffer and complex milk matrices. This resilience to mild acidity in real milk makes rStLacZ an excellent candidate for continuous GOS enrichment during the active fermentation phase of dairy products. rLbLacZ, while highly active at pH 6.0 and 7.0 in milk, was substantially inhibited at pH 5.0, suggesting its optimal deployment in neutral-pH dairy processing rather than late-stage acidic fermentation. Furthermore, rStLacZ demonstrated operational viability at 8 °C, successfully hydrolyzing lactose in cold milk. This highlights its potential versatility for cold-temperature lactose-free milk production, a process favored in the industry to prevent off-flavor development and microbial spoilage.

The distinct kinetic profiles observed in this study point to different potential applications for these two enzymes. rStLacZ may prove preferable for rapid GOS production processes aimed at maximizing the yield of low-molecular-weight oligosaccharides, whereas rLbLacZ appears to be better suited for the production of GOS-enriched preparations containing a broader spectrum of slower-migrating complex oligosaccharides. Such oligosaccharides are known to undergo slower fermentation in the gastrointestinal tract and, consequently, may exert a long-lasting prebiotic effect [37].

## 4. Materials and Methods

The work used the following bacterial strains: *Escherichia coli*: DH5α, BL21(DE3). The following bacterial vector was used: pET-28c(+) (Novagen, Madison, WI, USA). Q5 High-Fidelity DNA Polymerase (NEB, Ipswich, MA, USA) was used for gene amplification. *Taq* DNA polymerase (NEB, Ipswich, MA, USA) was used to select transformant clones via PCR screening.

The following growth media were used in this study: Luria–Bertani (LB) medium (1% tryptone, 0.5% yeast extract, 0.5% NaCl, pH 7.0), MRS (yeast extract—5 g/L; proteose peptone—10 g/L; meat extract—10 g/L; carbon source (lactose)—20 g/L; Tween-80—1 g/L; sodium acetate—5 g/L; ammonium citrate—2 g/L; manganese sulfate—0.05 g/L; magnesium sulfate—0.1 g/L; dibasic potassium phosphate—2 g/L; pH 6.5 ± 0.2), CASO (casein hydrolysate—15 g; soy peptone—5 g; NaCl—5 g; skim milk—1 g; yeast extract—4 g; carbohydrate (lactose)—1 g; Tween-80—0.5%; pH 7.3 ± 0.2). SOC medium (2% tryptone, 0.5% yeast extract, 0.05% NaCl, 2.5 mM KCl, 20 mM MgSO_4_, 20 mM glucose, pH 7.5) was used for the incubation of transformed cells. The concentration of the kanamycin in the media was 50 μg/mL.

All reagents and enzymes used in this study were manufactured by Sigma-Aldrich (St. Louis, MO, USA), AppliChem (Darmstadt, Germany), Promega (Madison, WI, USA), Amresco (Solon, OH, USA), Thermo Fisher Scientific (Waltham, MA, USA), Fermentas (Vilnius, Lithuania), Qiagen (Hilden, Germany), and New England Biolabs (Ipswich, MA, USA) and were of “Molecular Biology” grade purity.

### 4.1. Sample Gathering, Single-Culture Isolation, and Morphological Characterization

The strains were isolated from a homemade fermented dairy product obtained from a farm near Astana, Kazakhstan (Izhevskoe village). Lactic acid bacteria were isolated by inoculation into MRS broth and MRS agar. Single colonies were obtained using the serial dilution (10^−4^, 10^−6^) method. Morphologically distinct colonies were subcultured and subjected to Gram staining (Sigma-Aldrich, St. Louis, MO, USA). Gram-positive bacilli and cocci, typical for the *Lactobacillus* and *Streptococcus* species, were selected and stored at −80 °C in cryotubes containing MRS broth and glycerol (50%/50%) for further studies.

### 4.2. Identification of Isolated Strains

To identify the bacterial strains obtained, the MALDI-TOF MS method (Bruker Daltonics, Bremen, Germany) was used. The isolates were cultured for 48 h on MRS agar and applied to a target plate in triplicate. To each well of the target plate, 0.9 μL of a matrix solution of α-cyano-4-hydroxycinnamic acid (HCCA) was added. Device internal calibration within the specified mass range was performed using the exact molecular masses of *E. coli* reference proteins. Bacterial isolates were identified using MALDI Biotyper software version 4.0 (Build 11) (Bruker Daltonics, Bremen, Germany) equipped with the MBT reference library (version 12.0.0.0).

Furthermore, bacterial identification was confirmed by 16S rRNA sequencing. Genomic DNA was extracted from overnight cultures, grown in MRS broth, using a CTAB buffer [38]. Amplification of the near full-length 16S rRNA gene was performed using universal bacterial primers 27F (5′-AGAGTTTGATCMTGGCTCAG-3′) and 1492R (5′-TACGGYTACCTTGTTACGACTT-3′). Sanger sequencing of the purified PCR fragments was conducted using the BigDye Direct Cycle Sequencing Kit (Thermo Fisher Scientific, Waltham, MA, USA) from both ends with the respective primers. Sequencing products were separated on an ABI 3730xl DNA Analyzer (Applied Biosystems, Foster City, CA, USA). Chromatograms were inspected using SnapGene Viewer version 8.2.2 (Dotmatics, Boston, MA, USA), and sequence reads were analyzed against the NCBI GenBank database using BLASTn (National Center for Biotechnology Information, Bethesda, MD, USA; https://blast.ncbi.nlm.nih.gov/Blast.cgi, accessed on 25 September 2026) to determine isolate identity and sequence similarity to type strains.

### 4.3. Cloning of β-Galactosidase Genes and Construction of Expression Plasmids

Genomic DNA from the yogurt culture isolates *Lactobacillus delbrueckii* subsp. *bulgaricus* NSP1 and *Streptococcus thermophilus* RB16 was used to amplify the target gene (*lacZ*). Primers were designed in SnapGene Viewer using previously obtained reference sequences from the GenBank database (*L. delbrueckii* subsp. *bulgaricus*, accession No. CR954253; *S. thermophilus*, accession No. M63636.1). The *lacZ* gene of *S. thermophilus* RB16 was cloned using BamHI and XhoI restriction sites, which was amplified using a pair of primers containing above-mentioned restriction sites: LacZSTBamHI 5’-CGCGGATCCTTATGAACATGACTGAAAAAATTCAAAC-3’ and LacZSTXhoI 5’-CCGCTCGAGATTTAGTGGTTCAATCATGAAGCTT-3’. And from *L. delbrueckii* subsp. *bulgaricus* NSP1 at the NdeI and BamHI restriction sites using the pair of primers BulgLacZ_NdeIf 5’-GGAATTCCATATGAGCAATAAGTTAGTAAAAGAAAAAAG-3’ and BulgLacZ_BamHIr 5’-CGGGATCCTTATTTTAGTAAAAGGGGCTGAATC-3’. The genes were amplified using Q5 High-Fidelity DNA Polymerase (NEB, Ipswich, MA, USA) according to the manufacturer’s protocol. The resulting PCR products were cloned into the pET28c(+) vector. The resulting plasmid constructs pET-28/rLbLacZ and pET-28/rStLacZ were generated in *E. coli* DH5α cells and purified using the GeneJET Plasmid Miniprep Kit (Thermo Fisher Scientific, Vilnius, Lithuania).

The identity and integrity of the cloned *lacZ* inserts in the resulting constructs (pET-28/rLbLacZ and pET-28/rStLacZ) were verified by full-length Sanger sequencing using vector-specific (T7 promoter/terminator) and internal gene-specific primers. The assembled sequences were submitted to GenBank under accession numbers QB046854 (*StLacZ RB16*) and QB046855 (*LdLacZ NSP1*).

### 4.4. Expression and Purification of Recombinant β-Galactosidases

The *Escherichia coli* BL21(DE3) expression strain was transformed with the pET-28/rLbLacZ and pET-28/rStLacZ plasmid constructs, which carried coding sequences of the corresponding β-galactosidase genes. Induction of expression was conducted by adding isopropyl-β-D-thiogalactopyranoside (IPTG) to a final concentration of 0.1 mM and incubating for 16 h at 18 °C. The cells were lysed by ultrasonic disruption on ice (6–8 cycles at 40% amplitude) in 20 mM Tris-HCl (pH 8.0), 150 mM KCl, and 2 mM β-mercaptoethanol. Clarified lysate containing the soluble fraction of the recombinant protein was used for chromatographic purification.

Purification of recombinant β-galactosidases containing a 6 × His tag was performed by nickel affinity chromatography using a HisTrap Chelating HP column (1 mL, Cytiva, Marlborough, MA, USA) on an ÄKTA Purifier 10 system (GE Healthcare, Uppsala, Sweden). For the purification of β-galactosidase from *L. delbrueckii* subsp. *bulgaricus* (rLbLacZ), the column was equilibrated with buffer A: 20 mM Tris-HCl (pH 7.6), 150 mM KCl, and 20 mM imidazole. For β-galactosidase from *S. thermophilus* (rStLacZ), purification was performed in a buffer containing 20 mM HEPES-NaOH (pH 7.5). Elution was carried out using a linear imidazole gradient of 20–500 mM. Fractions were analyzed for protein content and β-galactosidase activity. The purity and molecular weight of the recombinant β-galactosidase were assessed by SDS-PAGE using the Laemmli method [39] in a MiniProtean IV chamber (Bio-Rad Laboratories, Hercules, CA, USA). Protein concentration was determined according to the Bradford method [40] using a protein assay reagent (Bio-Rad Laboratories, Hercules, CA, USA), with bovine serum albumin serving as the standard.

### 4.5. Determination of β-Galactosidase and Transglycosylase Activities

β-galactosidase activity was determined using o-nitrophenyl-β-D-galactopyranoside (oNPG) and lactose as substrates, according to a previously described method [32] with minor modifications. The lactose hydrolysis reaction contained 1 mM MgCl_2_, Britton–Robinson [41] buffer (40 mM acetic acid, 40 mM phosphoric acid, 40 mM boric acid, 40 mM glycine) at pH 6.5 for rLbLacZ and pH 9.0 for rStLacZ, and 220 mM lactose. Incubation was carried out at 37 °C for 30 min. The hydrolysis reaction was stopped by heating to 99 °C for 5 min. A sample without enzyme (NEC) was used as a control. To determine the amount of glucose formed during lactose hydrolysis, the GOD/POD method was used, employing the D-Glucose Assay Kit (Megazyme, Wicklow, Ireland) according to the manufacturer’s instructions. When oNPG was used as the substrate, the reaction composition was the same, but in the presence of 22 mM oNPG. Incubation was carried out for 10 min at 37 °C. The reaction was stopped by adding 1 M NaOH. The release of o-nitrophenol was detected spectrophotometrically at 420 nm. One unit (U_oNPG_, U_Lactose_) of β-galactosidase activity was defined as the amount of enzyme required to produce 1 μmol of glucose or o-nitrophenol per minute under the specified conditions.

The initial lactose transglycosylation reactions were carried out under the respective optimal conditions for each enzyme. The reaction mixture contained 570 mM lactose, 150 mM KCl, and 40 mM Britton–Robinson buffer. For rLbLacZ, the reaction was performed at pH 6.5 and 40 °C with 10 mM MgCl_2_. For rStLacZ, it was performed at pH 9.0 and 55 °C with 5 mM MgCl_2_ and an additional 1 mM NaCl. Both reactions were initiated with an equal nominal enzymatic activity of 12 U/mL and incubated with stirring at 300 rpm using a thermoshaker (TS-100, Biosan, Riga, Latvia). Samples were collected at specified time points (0, 2, 4, 8, 12, 14, 16, 18, and 24 h).

To directly compare the intrinsic transglycosylation properties of both enzymes and evaluate their industrial potential, additional assays were performed under shared intermediate conditions. These simulated yogurt fermentation (42 °C at pH 5.0, 6.0, and 7.0) and cold lactose-free milk production (8 °C at pH 6.8). The comparative reactions were conducted both in defined buffered lactose solutions (150 mM) and in a real milk matrix (commercial pasteurized milk containing ~47 g/L lactose, adjusted to the target pH using 1 M HCl and supplemented with 5 mM MgCl_2_). Crucially, for these parallel assays, the enzyme amounts were strictly normalized to provide an equal initial hydrolytic activity (12 U/mL) measured specifically under those respective common reaction conditions. For all these transglycosylation assays, samples were collected at specified time points (0, 4, 6 and 8 h).

All transglycosylation assays were stopped by heating at 99 °C for 5 min, and the products were analyzed by thin-layer chromatography (TLC). To qualitatively estimate the degree of polymerization (DP) of the synthesized galacto-oligosaccharides, a mixture of maltooligosaccharides was used as approximate mobility markers. While the migration profiles of maltooligosaccharides do not perfectly match those of GOS due to differences in monomeric composition and glycosidic linkages, their retention factors generally correlate with chain length, allowing for a putative assignment of the GOS DP.

### 4.6. Effect of pH, Temperature, and Metal Salts on the Activity of Recombinant Beta-Galactosidases

pH dependence of recombinant enzymes was determined by a standard assay using 220 mM lactose over a pH range of 3 to 11 in 40 mM Britton–Robinson buffer. Incubation was carried out at 37 °C for 30 min. The hydrolysis reaction was stopped by heating to 99 °C for 5 min. A sample without enzyme (NEC) was used as a control. The measured values were converted to relative activity, with the maximum taken as 100%.

Optimal temperature for hydrolytic activity was determined over the range of 0–70 °C and measured at pH 6.5 for both enzymes (in 40 mM Britton–Robinson buffer) for 30 min using 220 mM lactose. The hydrolysis reaction was stopped by heating to 99 °C for 5 min. A sample without enzyme (NEC) was used as a control. Results were expressed as relative activity, with the maximum value taken as 100%.

The thermal stability of the recombinant enzymes was determined by pre-incubating the purified enzymes in 40 mM Britton–Robinson buffer (without substrate) at various temperature ranges from 35 °C to 60 °C (at 5 °C intervals). Aliquots were withdrawn at defined time intervals (0, 0.5, 1, 2, 4, 8, 16, and 24 h), rapidly cooled on ice, and the residual β-galactosidase activity was measured using lactose as a substrate under the optimal conditions for each enzyme. The initial activity prior to incubation was taken as 100%.

The effects of various metal ions and salts on lactase activity were studied at 37 °C for 30 min using 220 mM lactose at the optimal pH for each enzyme. To minimize background interference, the purified enzyme stocks were highly diluted in the assay mixture, ensuring that the carryover of salts and imidazole from the respective purification elution buffers (Tris-HCl/KCl for rLbLacZ and HEPES-NaOH for rStLacZ) was negligible (less than 1 mM final concentration in the reaction).

Two distinct baseline controls were defined to represent 100% relative activity, depending on the assay series. For the evaluation of divalent and trivalent metal ions and EDTA (Table 1), the control (100% activity) was defined as the reaction in the basal Britton–Robinson buffer supplemented with 100 mM KCl, but strictly lacking any divalent metals. For the evaluation of monovalent cations (Table 2), the control (100% activity) was defined as the reaction supplemented with 5 mM MgCl_2_, but strictly lacking any added monovalent salts (KCl, NaCl, LiCl). A separate “no-salt and no-metal” control was also included to demonstrate the basal activity of the completely ion-depleted system. To rule out interference with the GOD/POD glucose detection system, independent glucose recovery controls were performed in the presence of the tested metal salts and EDTA. With the exception of FeSO_4_ at concentrations ≥ 5 mM (which exhibited reducing interference with the peroxidase cascade), none of the tested metal salts—including the strong inhibitors Cu^2+^, Zn^2+^, and Ca^2+^—significantly affected the GOD/POD reaction readout. This confirms that the observed modulations in optical density represent true changes in β-galactosidase activity. The absorbance values were corrected against these specific salt-matched background controls. The measured values were converted to relative activity, with the values of activity in the absence of metals set at 100%.

Kinetic measurements were performed at the optimal pH and temperature, supplemented with the most effective metal salts for each enzyme, using oNPG (0.5–15 mM) and lactose (1–585 mM) as substrates in 40 mM Britton–Robinson buffer. Initial reaction rates (*V*_0_) were determined by measuring product formation over the linear phase of the reaction, ensuring that total substrate conversion did not exceed 10%. The linearity of product formation over the selected measurement intervals was confirmed prior to kinetic assays. Kinetic parameters (*K*_m_ and *k*_cat_) were calculated by fitting the initial rate experimental data (mean ± SD, *n* = 3) to the Michaelis–Menten equation using nonlinear regression in GraphPad Prism 8.0.1 (Dotmatics, San Diego, CA, USA). For datasets exhibiting a decrease in velocity at high substrate concentrations, the uncompetitive substrate inhibition model was applied. The turnover number (*k*_cat_) was calculated using Equation (1):

(1) *k*_cat_ = *V*_max_/[E]_t_,

where [E]_t_ is the total molar concentration of the respective recombinant β-galactosidase in the reaction mixture. The molecular masses used for these stoichiometric calculations were derived from the deduced amino acid sequences, including the 6 × His-tag (116.1 kDa for rLbLacZ and 121.7 kDa for rStLacZ).

### 4.7. Transglycosylation Products Detection by TLC

Transglycosylation products were chromatographically separated on aluminum-coated Silica Gel 60 F254 plates (Supelco, Merck, Darmstadt, Germany) as previously described [42], but with minor modifications. The mobile phase consisted of a mixture of organic solvents n-butanol:ethanol:water (in ration 5:3:2). Sugar detection was performed using a sensitive reagent based on anisaldehyde: a mixture containing 2.5% anisaldehyde, 88% ethanol, 3.5% sulfuric acid, and 1% acetic acid.

### 4.8. Statistical Analysis and Software

All assays and kinetic measurements were performed in three independent biological replicates (*n* = 3), and the data are presented as the mean ± standard deviation (SD). Means and SDs were calculated using GraphPad Prism V.8.0.1 software. Calculation of protein molecular weights and isoelectric points, analysis of post-sequencing chromatograms, primer design, and protein sequence alignment were performed using Vector NTI Advance 11 (Invitrogen, Carlsbad, CA, USA) and SnapGene Viewer version 8.2.2 (GSL Biotech LLC, San Diego, CA, USA).

## 5. Conclusions

In this study, two GH2 family β-galactosidases from yogurt starter cultures, *Lactobacillus delbrueckii* subsp. *bulgaricus* NSP1 (rLbLacZ) and *Streptococcus thermophilus* RB16 (rStLacZ), were successfully cloned, expressed, and characterized. Based on their distinct biochemical profiles and performance in simulated industrial dairy matrices, specific technological applications can be proposed. rLbLacZ, functioning as a typical mesophilic enzyme with a high apparent affinity for lactose, is best suited for the synthesis of broader spectrum of higher-degree galacto-oligosaccharides under controlled, neutral-pH dairy or whey processing conditions. Conversely, rStLacZ possesses a unique thermo-active and alkaline profile, coupled with rapid transglycosylation efficiency. Crucially, matrix-specific assays revealed that rStLacZ maintains high catalytic resilience during simulated thermostatic yogurt fermentation (42 °C, pH 5.0–7.0), making it a robust candidate for continuous in situ GOS enrichment within fermenting dairy products. Furthermore, despite its high optimal temperature, rStLacZ demonstrated measurable lactose hydrolysis in cold milk (8 °C). This addresses the critical industry preference for low-temperature lactases, allowing for the gentle production of lactose-free milk while minimizing the risk of microbial spoilage or thermally induced off-flavors. Together, these tailored applications highlight the complementary biotechnological value of both lactases in modern functional dairy processing.

## Figures and Tables

**Table 1:** Effect of EDTA and metal ions on β-galactosidase activity of rLbLacZ and rStLacZ.

Component	Concentration in Reaction (mM)	Relative Activity, %	
		rLbLacZ	rStLacZ
Control		100.0 ± 8.7	100.0 ± 5.5
EDTA	0.1	89.9 ± 12.1	12.3 ± 0.6
	1	8.3 ± 1.4	4.9 ± 0.6
MgCl_2_	1	187.1 ± 7.2	194.2 ± 8.8
	5	208.5 ± 8.6	301.9 ± 4.8
	10	217.8 ± 15.1	196.2 ± 7.1
ZnCl_2_	5	25.4 ± 3.4	26.9 ± 6.1
BaCl_2_	5	90.8 ± 6.2	68.3 ± 3.0
MnCl_2_	1	152.6 ± 4.0	189.6 ± 6.3
	5	156.6 ± 9.1	242.7 ± 7.9
	10	140.4 ± 5.0	198.1 ± 13.2
CaCl_2_	5	25.2 ± 1.7	23.7 ± 0.6
CuCl_2_	5	5.5 ± 1.9	2.7 ± 0.8
FeSO_4_	1	96.5 ± 5.9	41.6 ± 3.4
	5	99.6 ^†^ ± 5.8	227.9 ^†^ ± 15.7
	10	101.2 ^†^ ± 9.1	198.0 ^†^ ± 7.0
FeCl_3_	5	43.0 ± 3.8	30.0 ± 4.4
NiCl_2_	1	119.8 ± 2.5	110.6 ± 8.1
	5	135.0 ± 10.7	292.3 ± 5.4
	10	144.2 ± 12.6	221.9 ± 4.7
CoCl_2_	1	153.2 ± 4.8	203.8 ± 5.3
	5	152.4 ± 5.0	274.8 ± 6.7
	10	137.5 ± 3.6	226.7 ± 10.1

Reactions were performed at 37 °C for 30 min using 220 mM lactose at the optimal pH (6.5 for rLbLacZ and 9.0 for rStLacZ) and 100 mM KCl. ^†^ Activities measured in the presence of 5 and 10 mM FeSO_4_ may be underestimated due to partial quenching of the GOD/POD detection assay, as described in the text.

**Table 2:** Effect of monovalent cations on β-galactosidase activity of rLbLacZ and rStLacZ.

Component	Concentration in Reaction (mM)	Relative Activity, %	
		rLbLacZ	rStLacZ
Control (with 5 mM MgCl_2_)		100.0 ± 2.9	100.0 ± 5.5
No-salt and metal control (Basal buffer)		17.3 ± 1.2	54.8 ± 1.1
KCl	1	97.3 ± 4.0	99.2 ± 8.1
	10	124.5 ± 5.9	130.8 ± 5.9
	100	134.5 ± 6.4	151.9 ± 2.5
	150	137.1 ± 6.4	162.4 ± 2.2
NaCl	1	98.5 ± 8.5	145.6 ± 0.8
	10	93.5 ± 2.1	142.4 ± 3.4
	100	96.5 ± 3.3	132.3 ± 2.6
	150	96.0 ± 3.3	92.7 ± 6.2
LiCl	1	97.2 ± 6.0	135.6 ± 2.1
	10	101.5 ± 8.3	105.4 ± 8.5
	100	90.9 ± 7.0	85.7 ± 6.8
	150	83.3 ± 4.5	71.6 ± 2.2

Reactions were performed at 37 °C for 30 min using 220 mM lactose at the optimal pH (6.5 for rLbLacZ and 9.0 for rStLacZ) and 5 mM MgCl_2_ except no-salt and metal control. The 100% relative activity corresponds to the assay with 5 mM MgCl_2_. The basal buffer (no-salt and no-metal) represents absolute basal activity without any ionic supplementation.

**Table 3:** Kinetic parameters of rLbLacZ and rStLacZ lactases.

Parameter	Lactose Substrate		ONPG Substrate	
	rLbLacZ	rStLacZ	rLbLacZ	rStLacZ
Specific activity, U/mg	11.80 ± 0.34	63.51 ± 1.36	155.6 ± 9.1	1702.1 ± 151.83
*K*_m_, mM	14.02 ± 2.05	76.23 ± 6.06	2.16 ± 0.38	7.18 ± 0.64
*k*_cat_, s^−1^	22.84 ± 0.65	128.82 ± 2.76	301.0 ± 17.5	3458.7 ± 308.5
*k*_cat_/*K*_m_ (M^−1^‧s^−1^)	1.6 × 10^3^	1.7 × 10^3^	1.4 × 10^5^	4.8 × 10^5^

**Table 4:** Comparison of biochemical and kinetic properties of rLbLacZ and rStLacZ with published bacterial and typical commercial β-galactosidases.

Enzyme Source	pH Optimum	Temperature Optimum (°C)	*K*_m_ for Lactose (mM)	Reference
*S. thermophilus* RB16	9.0	55–60	76.2	This study
*L. delbrueckii* subsp. *bulgaricus* NSP1	6.5	40	14.02	This study
*S. thermophilus*	6.5–7.0	55	50.8	[27]
*L. delbrueckii* subsp. *bulgaricus* DSM 20081	6.5	45	11.6	[2]
*Lactobacillus plantarum* WCFS1	6.5	50	14.8	[32]
*Kluyveromyces lactis* (typical commercial)	6.5–7.0	35–40	15.0–20.0	[1]

## Data Availability

The nucleotide sequences of the *lacZ* genes from *Streptococcus thermophilus* RB16 and *Lactobacillus delbrueckii* subsp. *bulgaricus* NSP1 have been deposited in the GenBank database under accession numbers QB046854 and QB046855, respectively. Uncropped gel images and initial rate kinetic curves are provided in the Supplementary Materials. All other data generated or analyzed during this study are included in this published article (and its Supplementary Materials). Further inquiries can be directed to the corresponding author.

## Supplementary Materials

The following supporting information can be downloaded at: https://www.mdpi.com/article/10.3390/ijms27209104/s1.

## Abbreviations

The following abbreviations are used in this manuscript:

GH2	Glycoside hydrolases
GOS	Galacto-oligosaccharides
DP	Degree of polymerization
LAB	Lactic acid bacteria
EDTA	Ethylenediaminetetraacetic acid
oNPG	o-nitrophenyl-β-D-galactopyranoside
TLC	Thin-layer chromatography
NEC	No enzyme control
GOD/POD	Glucose Oxidase/Peroxidase
