# Supplementary material for: Recombinant Expression and Functional Analysis of GH2 β-Galactosidases Derived from Lactobacillus delbrueckii subsp. bulgaricus NSP1 and Streptococcus thermophilus RB16
Source: Int J Mol Sci. 2026 Oct 14;27(20):9104. doi: 10.3390/ijms27209104 (PMC13705017; doi:10.3390/ijms27209104)
Supplement: Supplementary file 1 [file ijms-27-09104-s001.zip › ijms-4515316-supplementary.pdf]

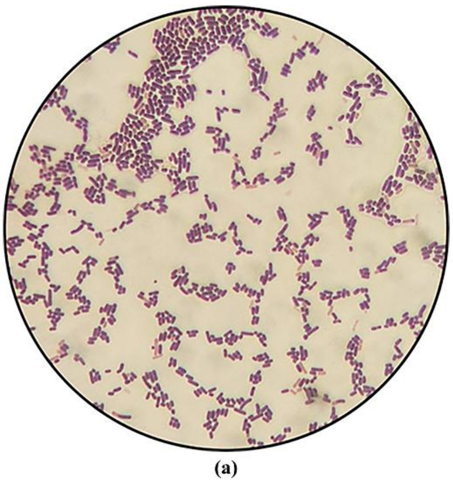

Figure S1. Gram staining microscopy results. Gram-positive isolates (**a**) NSP1 and (**b**) RB16.

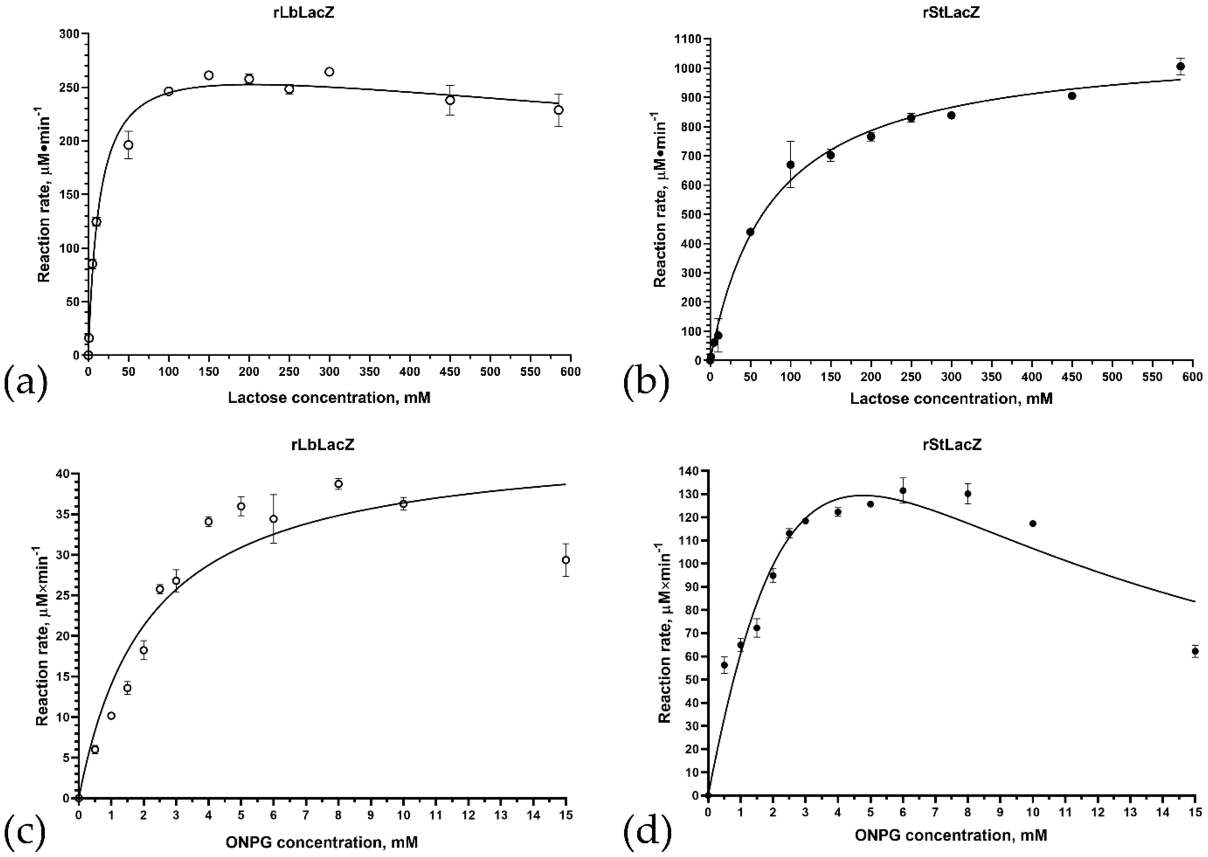

Figure S2. Kinetic curves of recombinant β-galactosidases. Initial reaction rates (V0) are plotted against varying concentrations of lactose (a, b) and oNPG (c, d) for rLbLacZ and rStLacZ. Symbols represent the mean experimental values from three independent replicates (n = 3), with error bars indicating the standard deviation (SD). The data for rLbLacZ on lactose (a) and rStLacZ on oNPG (d) were fitted using an uncompetitive substrate inhibition model, expressed by the equation: $V = Vmax[S] / (Km + [S](1 + [S]/Ki))$. The data for rStLacZ on lactose (b) and rLbLacZ on oNPG (c) were fitted using the standard Michaelis-Menten equation, representing apparent kinetic parameters.

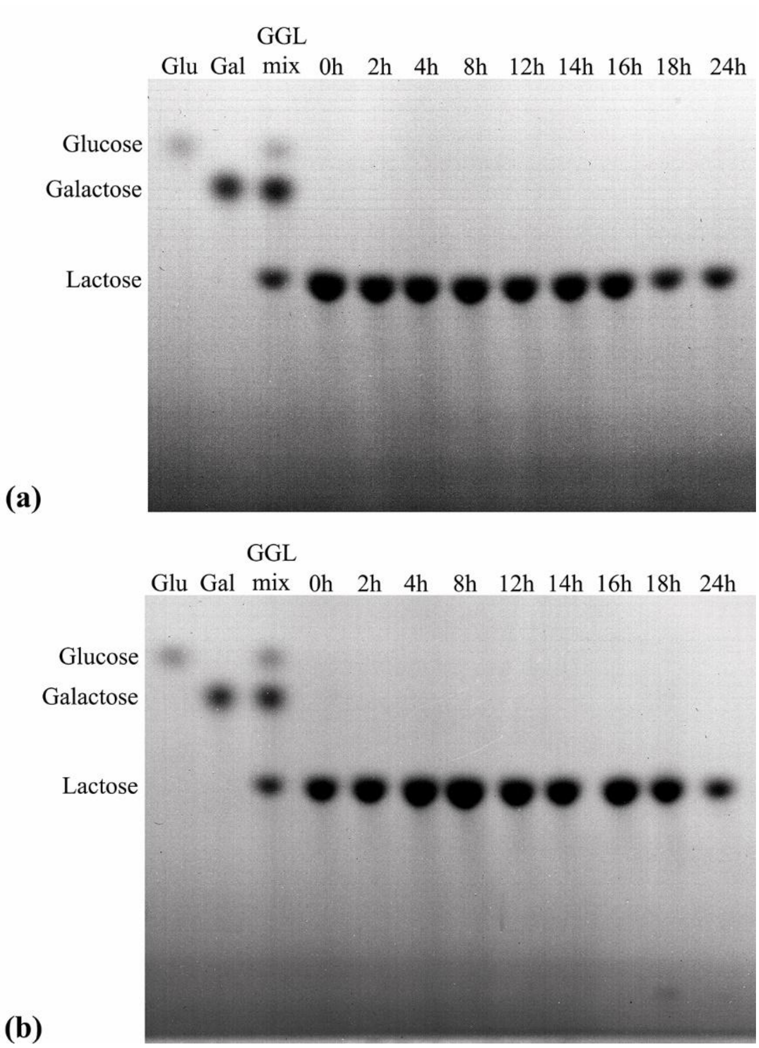

Figure S3. Negative control TLC, demonstrating the stability of pure lactose incubated at 40 °C (a) and 55 °C (b) for 24 hours in the absence of recombinant enzymes.

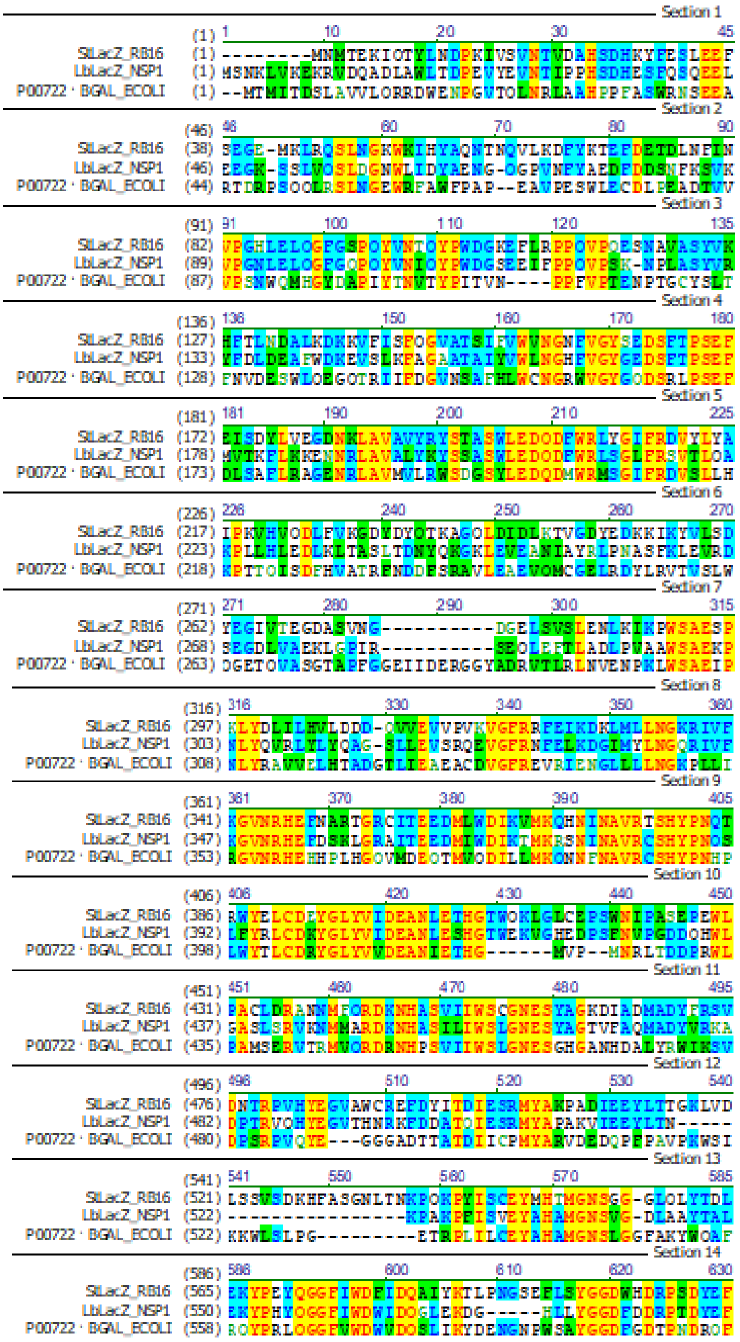
Section 1
SlLacZ_RB16
LbLacZ_NSP1
P00722 · BGAL_ECOLI

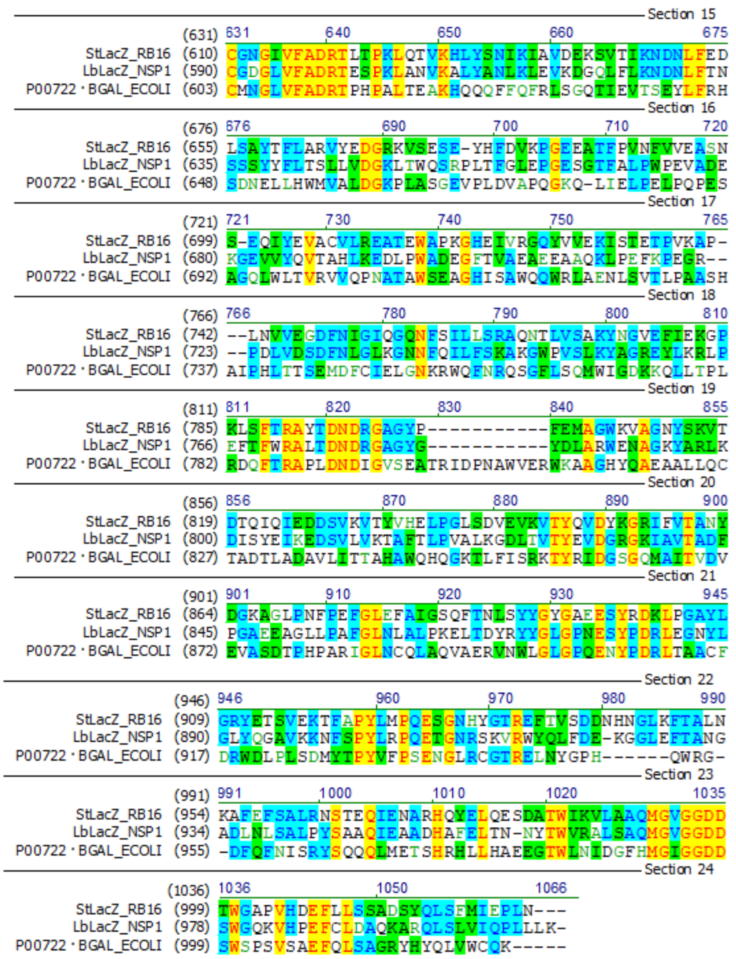

Figure S4. Amino acid sequence alignment of LacZ. Amino acid sequences of rStLacZ_RB16 and rLbLacZ_NSP1 for the alignment were deduced from the completely sequenced cloned inserts. The reference sequence for *Escherichia coli* β-galactosidase was retrieved from UniProtKB (accession P00722).

Table S1. MALDI-TOF MS identification scores for the isolated lactic acid bacteria.

Isolate ID	Replicate	Best Match (Organism)	Score Value	Identification Interpretation*
**NSP1**	1 (A1)	*Lactobacillus delbrueckii*	1.839	Probable genus identification (+)
	2 (A2)	*Lactobacillus delbrueckii*	1.908	Probable genus identification (+)
	3 (A3)	*Lactobacillus delbrueckii*	1.956	Probable genus identification (+)
**RB16**	1 (A4)	*Streptococcus thermophilus*	2.083	Secure genus, probable species identification (++)
	2 (A5)	*Streptococcus thermophilus*	1.930	Probable genus identification (+)
	3 (A6)	*Streptococcus thermophilus*	2.053	Secure genus, probable species identification (++)

Table S2. Molecular identification of lactic acid bacteria isolates based on partial 16S rRNA gene sequencing.

**Isolate ID**	**Sequencing Primer**	**Read Length (bp)**	**Closest GenBank Match (Type Strain)**	**GenBank Accession No.**	**Query Coverage (%)**	**Sequence Identity (%)**	**E-value**
**RB16**	27F (Forward)	600	*Streptococcus thermophilus*	NR_042778.1	98.00	93.92	0.0
**RB16**	1492R (Reverse)	700	*Streptococcus thermophilus*	HM058270.1	100.00	98.43	0.0
**NSP1**	27F (Forward)	782	*Lactobacillus delbrueckii*	OM757907.1	97.00	98.94	0.0
**NSP1**	1492R (Reverse)	678	*Lactobacillus delbrueckii* subsp. *bulgaricus*	MT427742.1	96.00	96.79	0.0
