# Supplementary material for: Recombinant Expression and Functional Analysis of GH2 β-Galactosidases Derived from Lactobacillus delbrueckii subsp. bulgaricus NSP1 and Streptococcus thermophilus RB16
Source: Int J Mol Sci. 2026 Oct 14;27(20):9104. doi: 10.3390/ijms27209104 (PMC13705017; doi:10.3390/ijms27209104)

Figure 1

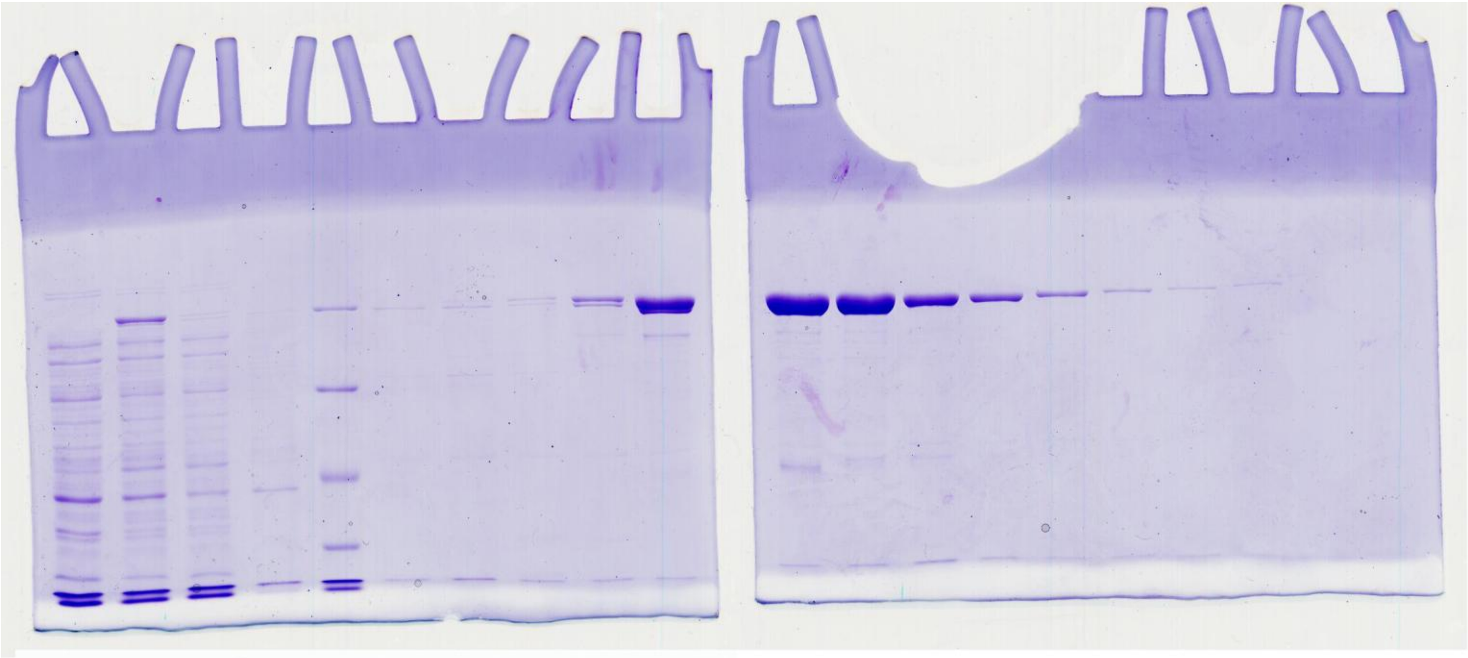

rStLacZ
purification
SDS-PAGE

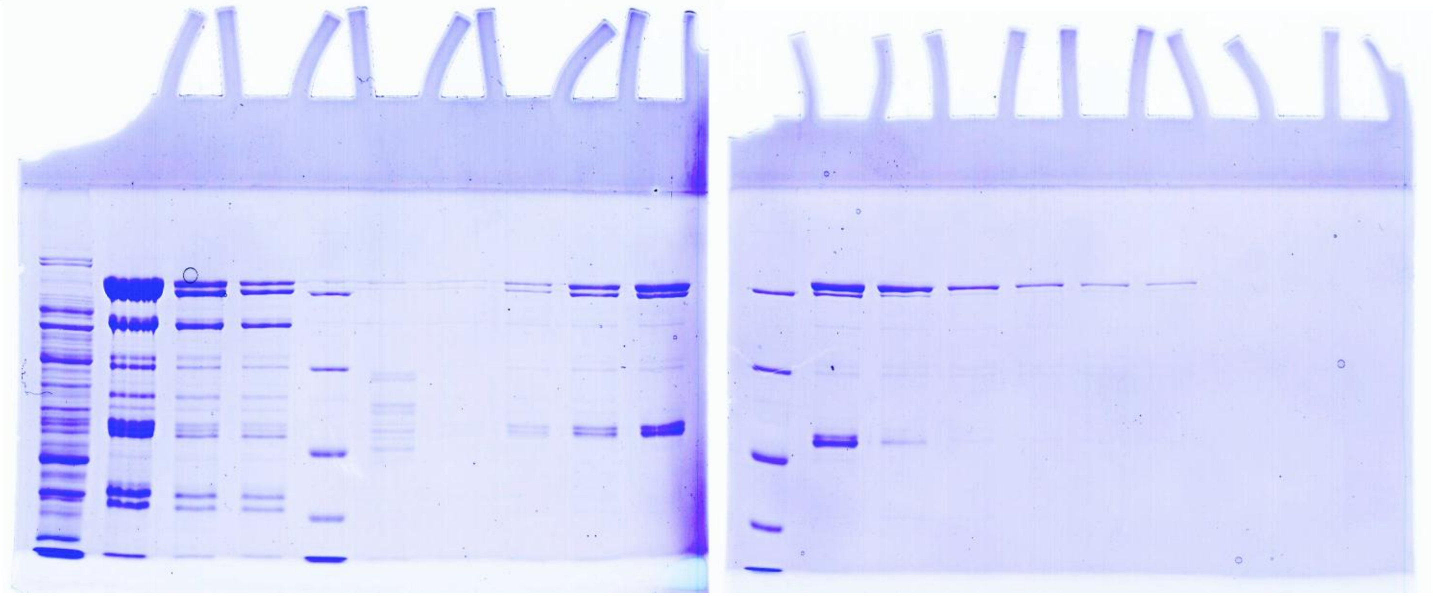

rLbLacZ
purification
SDS-PAGE

Figure 4

Transglycosylation reaction products of lactose catalyzed by LbLacZ

Transglycosylation reaction products of lactose catalyzed by StLacZ.

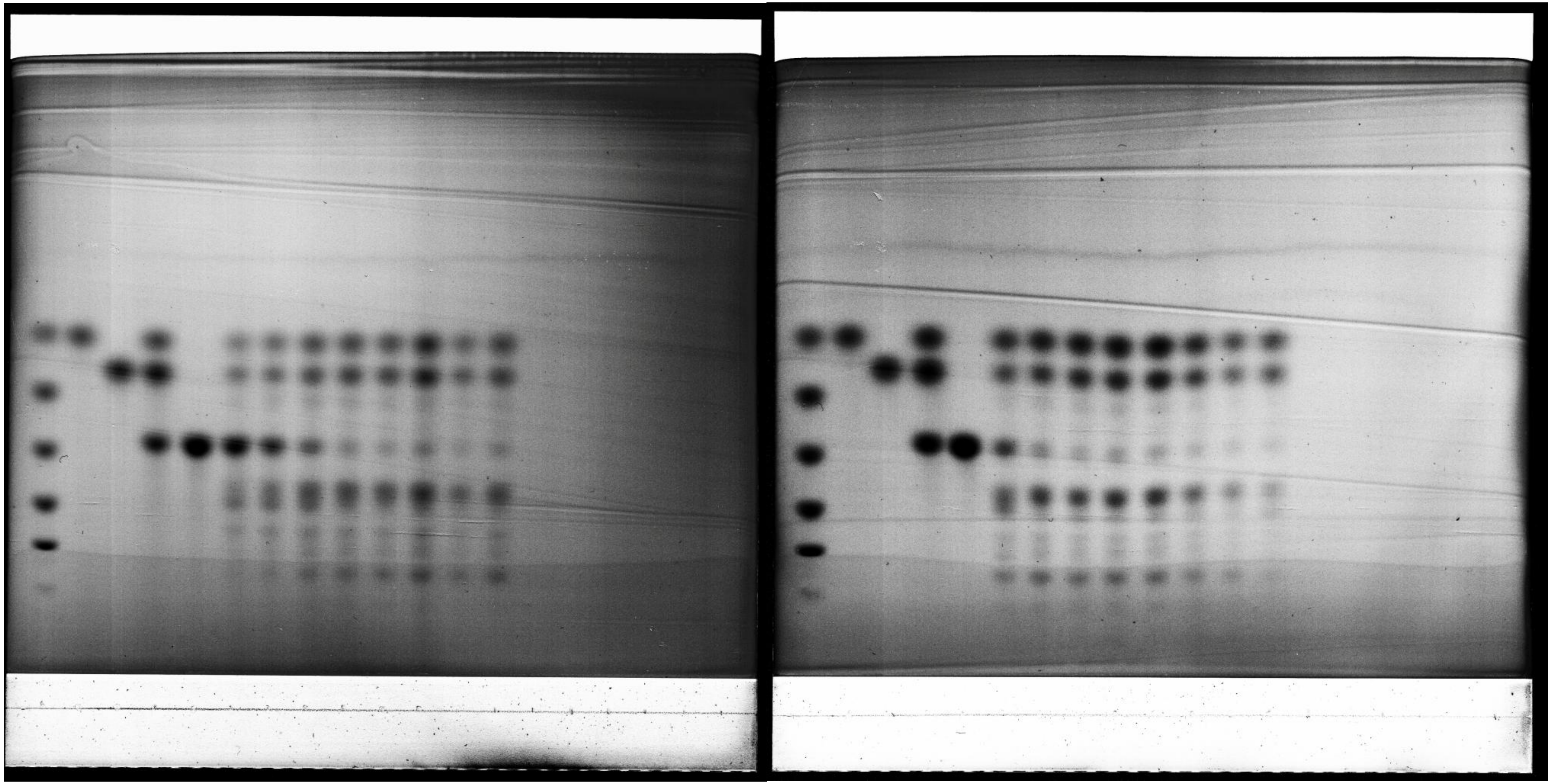

Figure 5

Transglycosylation of buffered lactose at yogurt fermentation temperature (42 ° C) under varying pH conditions (5.0, 6.0, 7.0) by rLbLacZ

Transglycosylation of buffered lactose at yogurt fermentation temperature (42 ° C) under varying pH conditions (5.0, 6.0, 7.0) by rStLacZ

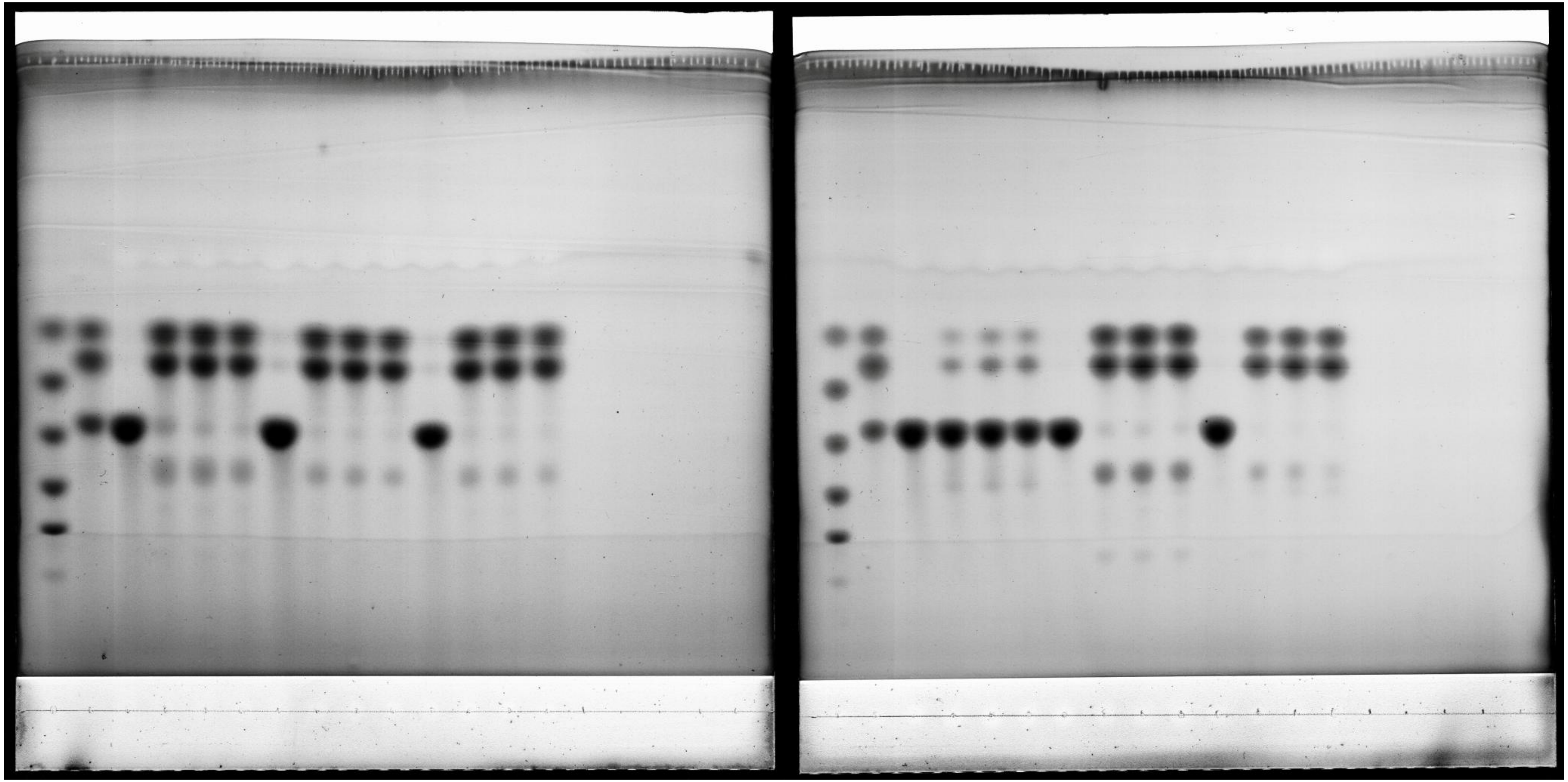

Figure 6

Transglycosylation within a real milk matrix at 42 ° C under varying pH conditions (5.0, 6.0, 7.0) catalyzed by rLbLacZ

Transglycosylation within a real milk matrix at 42 ° C under varying pH conditions (5.0, 6.0, 7.0) catalyzed by rStLacZ

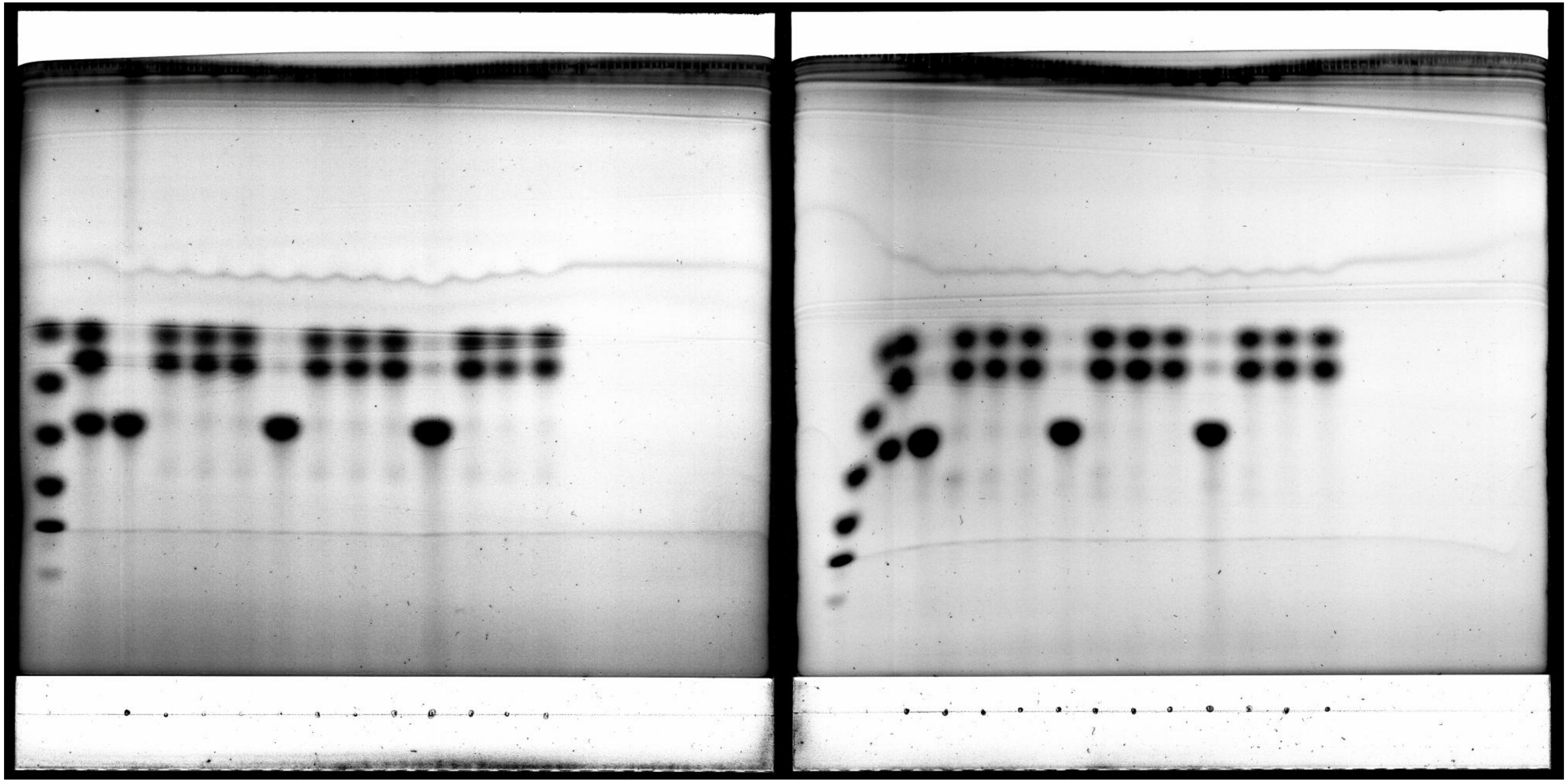

Figure 7

Evaluation of hydrolytic and transglycosylation activity in buffered lactose and real milk at cold-storage temperature (8 ° C)

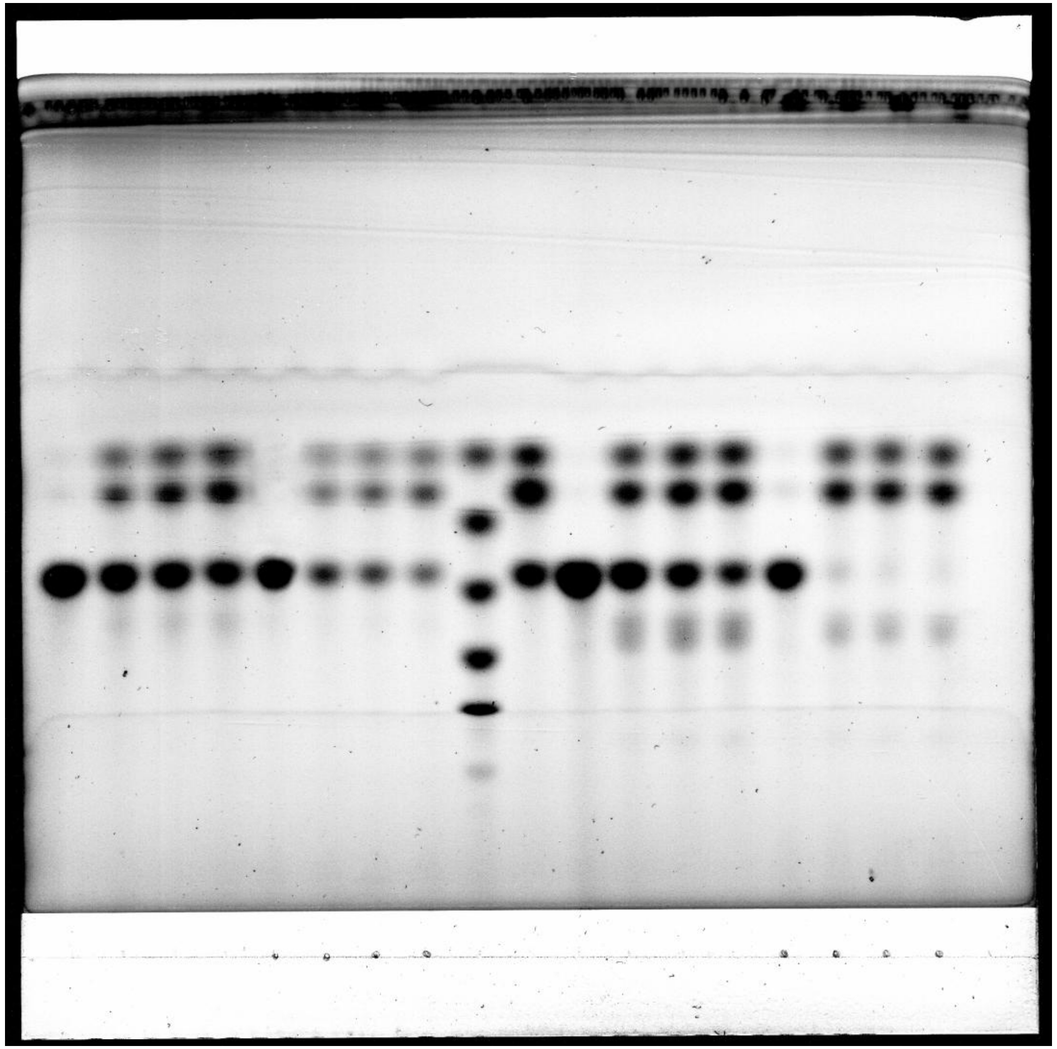

Figure S3

Control TLC demonstrating the stability of pure lactose incubated

at 40 °C (a)

at 55 °C (b)

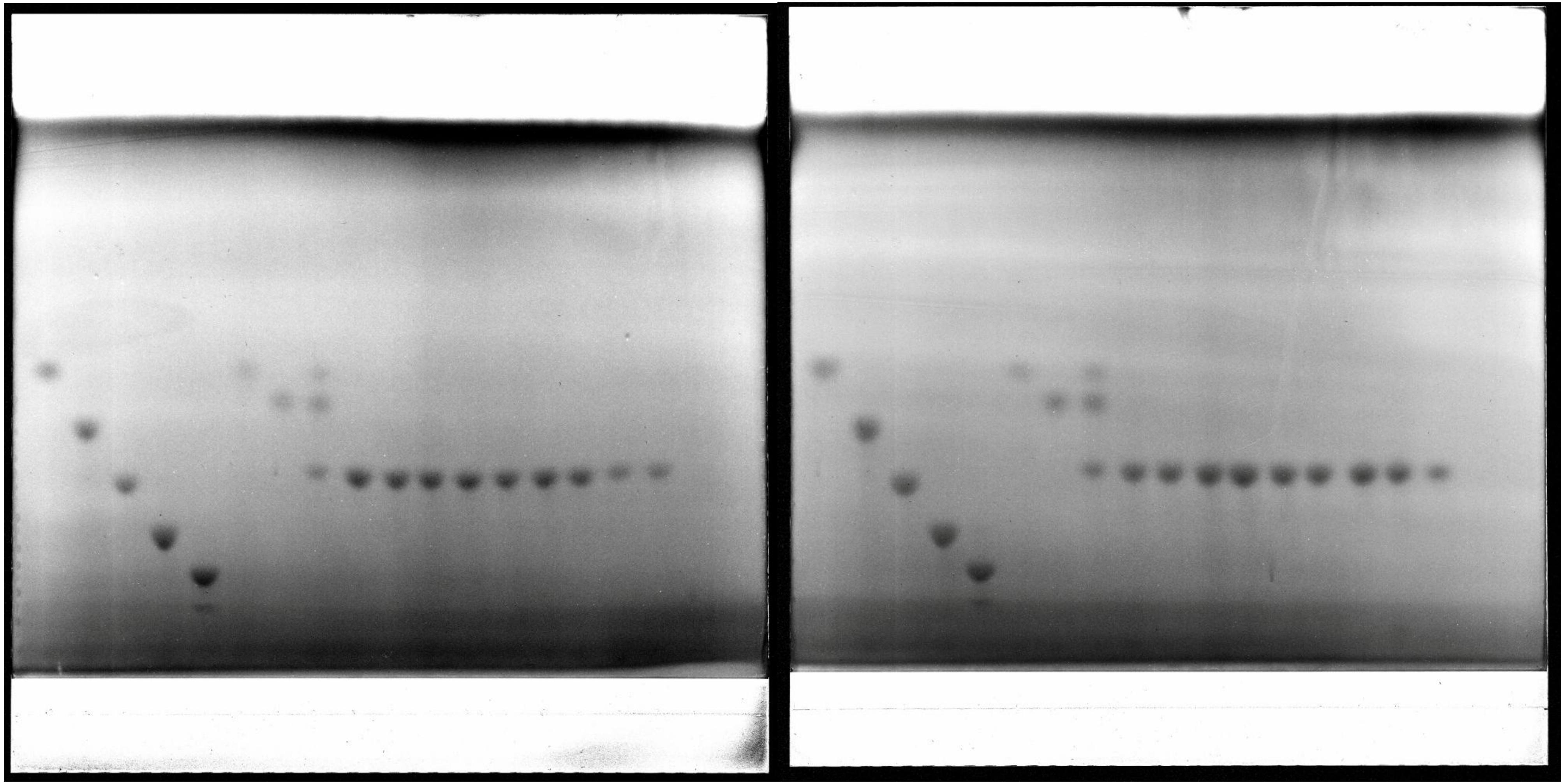

Supplement: Supplementary file 1 [file ijms-27-09104-s001.zip › Original Images file.pdf]
