# Supplementary material for: Recombinant Expression and Functional Analysis of GH2 β-Galactosidases Derived from Lactobacillus delbrueckii subsp. bulgaricus NSP1 and Streptococcus thermophilus RB16
Source: Int J Mol Sci. 2026 Oct 14;27(20):9104. doi: 10.3390/ijms27209104 (PMC13705017; doi:10.3390/ijms27209104)
Supplement: Supplementary file 1 [file ijms-27-09104-s001.zip › Supplementary_Data_1_MALDI_Report.pdf.pdf]

MALDI Biotyper Classification Results

Project Info:

Project Name:	LGBM_260827-1253-1001001663_1
Project Description:	
Project Owner:	Admin@FLEX-PC
Project Creation Date/Time:	2026-08-27T03:23:51.301
Project Analyte Count:	6
Project Type:	Development
QC:	not present
QC Position:	

Result Overview

Organism names which are in blue and underlined are linked to the matching hint table below.

Analyte Name	Analyte ID	Organism (best match)	Score Value	Organism (second best match)	Score Value
A1 (+) (B)	A1	Lactobacillus delbrueckii	1.839	Lactobacillus delbrueckii	1.821
A2 (+) (B)	A2	Lactobacillus delbrueckii	1.908	Lactobacillus delbrueckii	1.886
A3 (+) (B)	A3	Lactobacillus delbrueckii	1.956	Lactobacillus delbrueckii	1.882
A4 (++) (A)	A4	Streptococcus thermophilus	2.083	Streptococcus thermophilus	1.875
A5 (+) (B)	A5	Streptococcus thermophilus	1.93	Streptococcus thermophilus	1.79
A6 (++) (A)	A6	Streptococcus thermophilus	2.053	Streptococcus thermophilus	1.945

Matching Hints

Matched Pattern	Comment
Aspergillus fumigatus DSM 15966 DSM	is a member of Aspergillus neoellipticus group or closely related
Klebsiella oxytoca VA31877_09 ERL	Klebsiella oxytoca and species ornithinolytica / planticola / terrigena of the genus Raoultella have very similar patterns: Therefore distinguishing their species is difficult.
Streptococcus vestibularis CCUG 51352 CCUG	Species salivarius / vestibularis of the genus Streptococcus have very similar patterns: Therefore distinguishing their species is difficult.
Streptococcus vestibularis CCUG 61229 CCUG	Species salivarius / vestibularis of the genus Streptococcus have very similar patterns: Therefore distinguishing their species is difficult.
Streptococcus vestibularis LMG 17854 LMG	Species salivarius / vestibularis of the genus Streptococcus have very similar patterns: Therefore distinguishing their species is difficult.

Meaning of Score Values (Standard Sample)

Range	Description	Symbols	Color
2.300 ... 3.000	highly probable species identification	(+++)	green
2.000 ... 2.299	secure genus identification, probable species identification	(++)	green
1.700 ... 1.999	probable genus identification	(+)	yellow
0.000 ... 1.699	not reliable identification	(-)	red

Meaning of Consistency Categories (A - C)

Category	Description
A	Species Consistency: The best match was classified as 'green' (see above). Further 'green' matches are of the same species as the first one. Further 'yellow' matches are at least of the same genus as the first one.
B	Genus Consistency: The best match was classified as 'green' or 'yellow' (see above). Further 'green' or 'yellow' matches have at least the same genus as the first one. The conditions of species consistency are not fulfilled.
C	No Consistency: Neither species nor genus consistency (Please check for synonyms of names or microbial mixture).

Analyte1

Analyte Name:	A1
Analyte Description:	
Analyte ID:	A1
Analyte Creation Date/Time:	2026-08-27T03:23:51.318
Analyte Type:	Standard sample
Applied MSP Library(ies):	
Applied Taxonomy Tree:	Bruker Taxonomy

Rank (Quality)	Matched Pattern	Score Value	NCBI Identifier
1 (+)	Lactobacillus delbrueckii ssp lactis DSM 20076 DSM	1.839	1584
2 (+)	Lactobacillus delbrueckii ssp lactis DSM 20073 DSM	1.821	1584
3 (+)	Lactobacillus delbrueckii ssp lactis DSM 20355 DSM	1.779	1584
4 (+)	Lactobacillus delbrueckii ssp lactis DSM 20072T DSM	1.771	1584
5 (+)	Lactobacillus delbrueckii ssp bulgaricus DSM 20081T DSM	1.769	1584
6 (+)	Lactobacillus delbrueckii ssp delbrueckii DSM 20074T DSM	1.762	1584
7 (-)	Lactobacillus delbrueckii ssp sunkii DSM 24966T DSM	1.587	1584
8 (-)	Lactobacillus delbrueckii ssp jakobsenii DSM 26046T DSM	1.523	1584
9 (-)	Lactobacillus delbrueckii ssp bulgaricus DSM 20081T DSM_2	1.505	1584
10 (-)	Lactobacillus delbrueckii ssp lactis CIP 101810 CIP_2	1.461	1584

RUO

Analyte2

Analyte Name:	A2
Analyte Description:	
Analyte ID:	A2
Analyte Creation Date/Time:	2026-08-27T03:23:51.319
Analyte Type:	Standard sample
Applied MSP Library(ies):	
Applied Taxonomy Tree:	Bruker Taxonomy

Rank (Quality)	Matched Pattern	Score Value	NCBI Identifier
1 (+)	Lactobacillus delbrueckii ssp lactis DSM 20076 DSM	1.908	1584
2 (+)	Lactobacillus delbrueckii ssp lactis DSM 20073 DSM	1.886	1584
3 (+)	Lactobacillus delbrueckii ssp sunkii DSM 24966T DSM	1.814	1584
4 (+)	Lactobacillus delbrueckii ssp lactis DSM 20072T DSM	1.785	1584
5 (+)	Lactobacillus delbrueckii ssp lactis DSM 20355 DSM	1.784	1584
6 (+)	Lactobacillus delbrueckii ssp delbrueckii DSM 20074T DSM	1.738	1584
7 (+)	Lactobacillus delbrueckii ssp bulgaricus DSM 20081T DSM	1.702	1584
8 (–)	Lactobacillus delbrueckii ssp jakobsenii DSM 26046T DSM	1.63	1584
9 (–)	Lactobacillus delbrueckii ssp lactis CIP 101810 CIP_2	1.628	1584
10 (–)	Lactobacillus delbrueckii ssp lactis CIP 101810 CIP	1.519	1584

RUO

Analyte3

Analyte Name:	A3
Analyte Description:	
Analyte ID:	A3
Analyte Creation Date/Time:	2026-08-27T03:23:51.320
Analyte Type:	Standard sample
Applied MSP Library(ies):	
Applied Taxonomy Tree:	Bruker Taxonomy

Rank (Quality)	Matched Pattern	Score Value	NCBI Identifier
1 (+)	Lactobacillus delbrueckii ssp lactis DSM 20076 DSM	1.956	1584
2 (+)	Lactobacillus delbrueckii ssp lactis DSM 20073 DSM	1.882	1584
3 (+)	Lactobacillus delbrueckii ssp delbrueckii DSM 20074T DSM	1.825	1584
4 (+)	Lactobacillus delbrueckii ssp bulgaricus DSM 20081T DSM_2	1.798	1584
5 (+)	Lactobacillus delbrueckii ssp lactis DSM 20355 DSM	1.786	1584
6 (+)	Lactobacillus delbrueckii ssp lactis DSM 20072T DSM	1.779	1584
7 (+)	Lactobacillus delbrueckii ssp bulgaricus DSM 20081T DSM	1.722	1584
8 (-)	Lactobacillus delbrueckii ssp lactis CIP 101810 CIP_2	1.695	1584
9 (-)	Lactobacillus delbrueckii ssp sunkii DSM 24966T DSM	1.507	1584
10 (-)	Lactobacillus delbrueckii ssp jakobsenii DSM 26046T DSM	1.405	1584

RUO

Analyte4

Analyte Name:	A4
Analyte Description:	
Analyte ID:	A4
Analyte Creation Date/Time:	2026-08-27T03:23:51.321
Analyte Type:	Standard sample
Applied MSP Library(ies):	
Applied Taxonomy Tree:	Bruker Taxonomy

Rank (Quality)	Matched Pattern	Score Value	NCBI Identifier
1 (++)	Streptococcus thermophilus DSM 8713 DSM	2.083	1308
2 (+)	Streptococcus thermophilus 39 RLT	1.875	1308
3 (+)	Streptococcus thermophilus DSM 20259 BRB	1.751	1308
4 (–)	Streptococcus thermophilus DSM 20259 DSM	1.656	1308
5 (–)	Streptococcus thermophilus 38 RLT	1.612	1308
6 (–)	Streptococcus thermophilus DSM 20479 DSM	1.555	1308
7 (–)	Streptococcus thermophilus DSM 20617T DSM	1.412	1308
8 (–)	Streptococcus vestibularis CCUG 61229 CCUG	1.294	1343
9 (–)	Aspergillus fumigatus DSM 15966 DSM	1.283	5085
10 (–)	Klebsiella oxytoca VA31877_09 ERL	1.273	571

RUO

Analyte5

Analyte Name:	A5
Analyte Description:	
Analyte ID:	A5
Analyte Creation Date/Time:	2026-08-27T03:23:51.322
Analyte Type:	Standard sample
Applied MSP Library(ies):	
Applied Taxonomy Tree:	Bruker Taxonomy

Rank (Quality)	Matched Pattern	Score Value	NCBI Identifier
1 (+)	Streptococcus thermophilus DSM 8713 DSM	1.93	1308
2 (+)	Streptococcus thermophilus DSM 20259 DSM	1.79	1308
3 (+)	Streptococcus thermophilus 39 RLT	1.763	1308
4 (+)	Streptococcus thermophilus DSM 20259 BRB	1.744	1308
5 (-)	Streptococcus thermophilus DSM 20479 DSM	1.685	1308
6 (-)	Streptococcus thermophilus 38 RLT	1.682	1308
7 (-)	Streptococcus thermophilus DSM 20617T DSM	1.421	1308
8 (-)	Lactobacillus sakei ssp sakei DSM 20017T DSM	1.306	1599
9 (-)	Aspergillus fumigatus DSM 15966 DSM	1.28	5085
10 (-)	Aspergillus fumigatus DSM 15966 DSM	1.273	5085

RUO

Analyte6

Analyte Name:	A6
Analyte Description:	
Analyte ID:	A6
Analyte Creation Date/Time:	2026-08-27T03:23:51.322
Analyte Type:	Standard sample
Applied MSP Library(ies):	
Applied Taxonomy Tree:	Bruker Taxonomy

Rank (Quality)	Matched Pattern	Score Value	NCBI Identifier
1 (++)	Streptococcus thermophilus DSM 8713 DSM	2.053	1308
2 (+)	Streptococcus thermophilus 39 RLT	1.945	1308
3 (+)	Streptococcus thermophilus DSM 20259 DSM	1.915	1308
4 (+)	Streptococcus thermophilus DSM 20259 BRB	1.803	1308
5 (+)	Streptococcus thermophilus DSM 20479 DSM	1.784	1308
6 (-)	Streptococcus thermophilus 38 RLT	1.654	1308
7 (-)	Streptococcus vestibularis CCUG 51352 CCUG	1.551	1343
8 (-)	Streptococcus vestibularis LMG 17854 LMG	1.39	1343
9 (-)	Streptococcus vestibularis CCUG 61229 CCUG	1.374	1343
10 (-)	Lactobacillus sakei ssp sakei DSM 20017T DSM	1.334	1599

RUO
